# Prevalence of Frailty Among Chinese Community-Dwelling Older Adults: A Systematic Review and Meta-Analysis

**DOI:** 10.3389/ijph.2023.1605964

**Published:** 2023-08-01

**Authors:** Qi Zhou, Yao Li, Qiang Gao, Huiping Yuan, Liang Sun, Huan Xi, Wenbin Wu

**Affiliations:** ^1^ The Key Laboratory of Geriatrics, Beijing Institute of Geriatrics, Chinese Academy of Medical Sciences, Beijing Hospital/National Center of Gerontology of National Health Commission, Beijing, China; ^2^ Department of Thyroid-Breast-Hernia Surgery, Department of General Surgery, Beijing Hospital, National Center of Gerontology, Institute of Geriatric Medicine, Chinese Academy of Medical Sciences, Beijing, China; ^3^ Department of Scientific Research, Beijing Hospital, National Center of Gerontology, Institute of Geriatric Medicine, Chinese Academy of Medical Sciences, Beijing, China; ^4^ Department of Geriatrics, Beijing Hospital, National Center of Gerontology, Institute of Geriatric Medicine, Chinese Academy of Medical Sciences, Beijing, China

**Keywords:** frailty, community-dwelling, older adults, regional disparities, economic condition

## Abstract

**Objectives:** To systematically review the epidemiology of frailty in China, one of the world’s most populous countries, and to provide insightful guidance for countries to deal with fast population ageing.

**Methods:** Six electronic databases were searched until November 2022. Data from cross-sectional studies with a clear definition of frailty and a mean age ≥60 years were pooled using meta-analysis.

**Results:** 64 studies (*n* = 106,826 participants) from 23 (67.6%) of China’s provinces were included. The overall prevalence of frailty and prefrailty among older community dwellers was 10.1% (95% CI: 8.5%–11.7%) and 43.9% (95% CI: 40.1%–47.8%), respectively. Adults over 70 years, women, unmarried, living alone, and those with less education had higher odds of being frail. Furthermore, regional disparities in frailty were observed; people in rural areas or areas with worse economic conditions had a higher prevalence of frailty.

**Conclusion:** A great variation in frailty prevalence was observed between subgroups of older adults stratified by common risk factors. The Chinese government should pay more attentions to seniors at high risk and regions with a high prevalence of frailty.

## Introduction

Frailty is characterised by vulnerability to resist health stressors and is prevalent among older adults. It was associated with high incidences of hospitalisation, disability, dementia, and mortality [1–3] and poses enormous challenges to global healthcare systems. China’s healthcare systems face the same difficulty with high prevalence since the number of seniors has transcended 13.5% of the entire population. At the same time, inadequate eldercare can be provided by shrinking family size due to the one-child policy performed in the past 40 years. Therefore, understanding the prevalence of frailty is important for the Chinese government to prevent, intervene, and control disease development [4].

There is a relative lack of knowledge about the prevalence of frailty among Chinese seniors in the community. According to a systematic review conducted in 2019, the weighted prevalence of frailty and prefrailty among Chinese community-dwelling older adults was 10% and 43%, respectively [5]. Only five regions—Beijing, Hong Kong, Jinan, Langfang, and Taiwan—were pooled in this meta-analysis; the results may not accurately reflect the prevalence across China. China has 34 provinces or regions, among which there are great inequalities exist between developed and developing areas in healthcare use. Therefore, health issues, including the incidence of frailty, can be very different [6–8] between the regions. A national investigation showed a prevalence of frailty (7%). However, the study was conducted in 2011–2012, which cannot reflect the China’s frailty level in recent years [9].

Many factors, including age, sex, geography, living arrangement, marital status, and comorbidities, have affected the frail status, leading to a great variation in its prevalence [5, 10, 11]. Generally, frailty prevalence increases almost in multiples among individuals between 60 and 80 years; it is more common in women than in men; the frail ones usually suffer from over three chronic diseases. Additionally, there are more than ten ways to define frailty, including Fried Frailty Phenotype (FFP) [12], FRAIL scale (FRAIL) [13], Tilburg Frailty Indicator (TFI) [14], Rockwood’s Frailty Index (FI) [15], Edmonton Frailty Scale (EFS) [16], Vulnerable Elders Survey (VES-13) [17], and so on. These criteria, representing different conceptual frameworks, also complicate our understanding of the overall prevalence of frailty.

China launched the “Health China 2030” project in 2016, extensively promoting the progress of geriatric research [18]. Many frailty-related papers have been published in the past 5 years, making it possible to review the prevalence of frailty more comprehensively. Therefore, this systematic review and meta-analysis aimed to determine the pooled prevalence of frailty and document the characteristics of the prevalence stratified by factors such as diagnostic criteria, age, sex, urbanity, schooling time, living arrangement, and marital status.

## Methods

### Protocol

A systematic review of the literature was conducted in November 2022 following the Preferred Reporting Items for Systematic Review and Meta-Analysis (PRISMA) guidelines [19]. The protocol is registered and available at https://www.crd.york.ac.uk/PROSPERO with an ID of CRD42022344643.

### Search Strategy and Study Selection

We searched PubMed, EMBASE, Willey Online Library, Springer Link, China Knowledge Resource Integrated Database (CNKI), and Wanfang Database in English or Chinese. The search period was set from January 2011 to November 2022.

The medical subject heading (MeSH) and free text terms used were as follows: “Frailty (MeSH)” OR “frail*” AND “community (MeSH)” OR “community-dwelling” AND “China” OR “Chinese.” The reference lists of relevant and included articles were scrutinized. Additional relevant studies were manually identified from the references of the included studies or reviews.

Duplicates were removed after citations were identified. Two authors scanned the titles and abstracts of the studies for potential eligibility (QZ and YL). The full texts related to the inclusion criteria were further independently assessed by two authors (QZ and YL) and studies that met the exclusion criteria were discarded. Any discrepancies encountered during the selection process were solved through discussion.

The inclusion criteria were as follows: cross-sectional studies that reported the prevalence of frailty; participants with a mean age over 60 years; data collected from community-dwelling people residing in a Chinese area (including mainland China, Hong Kong, Macao, and Taiwan); an exact frailty diagnostic criterion can be found.

Exclusion criteria included studies defining frailty status using a continuous score, such as the FI definition, but without showing precise frailty prevalence; studies that screened or evaluated participants living in nursing homes, or were disease-specific samples (e.g., the entire sample had dementia or type 2 diabetes); randomised controlled trials, editorials, or conference abstracts.

### Data Extraction

The collected information was first author, publication year, location (province and cities), economic conditions of the related cities, urbanity (rural or urban areas), sample size, sampling strategy, time the study was performed, the proportion of females, mean age (or age range), language, frailty criteria, the prevalence of prefrailty and frailty. Likewise, the crude numbers of the participants stratified by sex, age, urbanity (rural/urban areas), schooling time (≤6 years vs. >6 years), and living arrangement (living alone vs. living with others) were also extracted from the studies. Two authors (QZ and HY) collected the data independently of the selected studies; any discrepancies were resolved by discussion.

An average of 10 years of gross domestic product (GDP) for cities was used to represent their economic condition. The raw GDP data were downloaded from the WIND Database by using a Financial Terminal. All data were stored in Microsoft Excel sheets.

### Assessment of Methodological Quality

Two authors (HY & LS) independently appraised each study using the Joanna Briggs Institute Critical Appraisal Checklist for Studies Reporting Prevalence Data (2020). The checklist contains nine questions representing nine dimensions of the study quality. A total score was calculated by the sum of “yes”, and larger scores mean the higher quality [20].

### Data Analysis

Meta-analysis was performed to estimate the pooled prevalence of frailty and prefrailty, and the pooled odds ratio (OR) and 95% confidence intervals (95% CI) for associations between frailty and potential risk factors (increased age, male, fewer years of schooling, living alone, and unmarried status). For studies that did not provide the ORs for the association between frailty and the risk factors, the crude numbers of frail participants in each subgroup (males and females) were extracted for the meta-analysis. A random effects model was chosen due to the recognition of substantial variability in the prevalence of frailty between individuals in different areas [21]. The pooled prevalence of frailty and prefrailty was also estimated in different subgroups stratified by diagnostic criteria (FFP vs. FRAIL vs. FI vs. TFI), age (60–69 vs. 70–79 vs.≥80), sex (male and female), urbanity (urban and rural areas), years of schooling (≤6 years and >6 years), living arrangement (living alone and living with others), marital status (married and unmarried), and geographical region.

The relationship between the prevalence of frailty and the economic condition was analysed by fitting a linear regression model or Mix-effected meta-regression. Cities were classified at high, middle, or low economic levels based on tertiles of the GDP.

Heterogeneity across the studies was examined using the chi-square test, and degrees of heterogeneity was quantified using the I^2^ statistic; I^2^ exceeding 75% was indicated high heterogeneity [22]. Sensitivity analysis was performed for pooled frailty prevalence using a leave-one-out strategy to identify potential outliers; subgroup analysis and meta-regression were further performed to explain the heterogeneity. Meta-regression was conducted with a mixed-effect model. Univariate meta-regression was first fitted to find a potential moderator for the prevalence of frailty, and then multivariate models were conducted by combining the potential moderators. Publication bias was assessed by visually inspecting funnel plots and confirmed by Egger tests.

All analyses were performed using R×64 (V4.1.2) with the package “meta” or the “metafor”; a two-sided *p*-value of less than 0.05 was considered statistically significant.

## Results

### Search Results and Characteristics of the Included Studies

**FIGURE 1:**
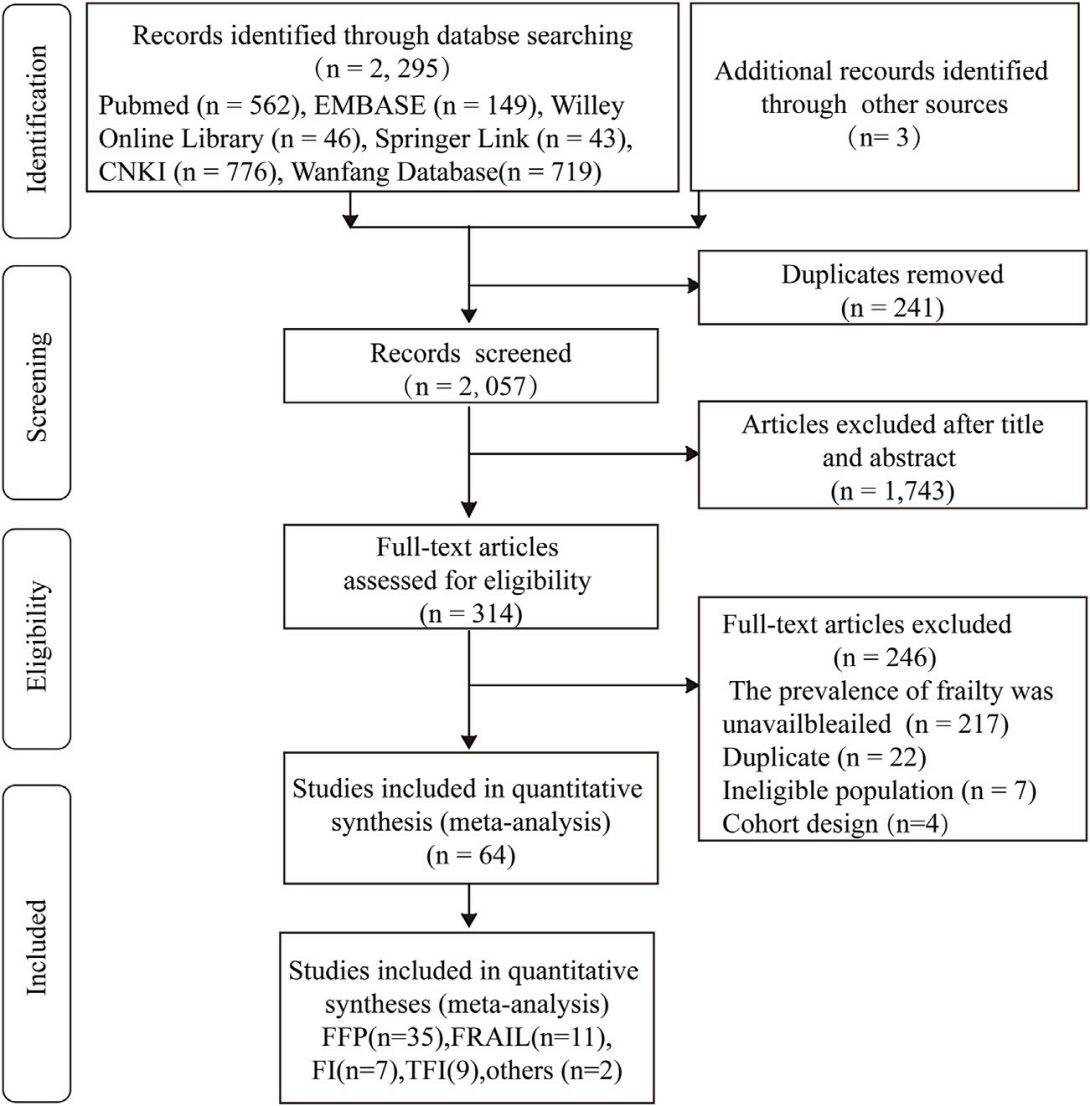
PRISMA flowchart for study selection. FFP, Fried frailty phenotype; FRAIL, the 5-term FRAIL scale; TFI, the Tilburg frailty indicator; FI, Rockwood’s frailty index (China, 2011–2022).

The flow chart of the literature search is shown in Figure 1. A total of 2,298 studies were identified, and 2,057 records were screened after removing duplicates. The title and abstract screening process excluded 1,743 papers, and the full texts of 314 studies were reviewed. A 246 studies were further removed due to missing data, duplicate participants, cohort study design, or an ineligible population. Finally, this review included 64 studies. 37 articles were written in Chinese, while 27 articles were English papers.

The characteristics of the included studies are shown in Supplementary Material S1. 23 Chinese provinces or regions with 106,826 participants were included in the present review. Most of the studies were focused on Shanghai (12 studies, 20,982 participants), Shandong Province (8 studies, 17,481 participants), and Beijing (8 studies, 11,330 participants); 57.8% of the studies (*n* = 37) were performed in the cities’ provincial capitals, while 17.2% of studies (*n* = 11) were conducted in only rural areas.

FFP (35 studies, 54.7%) was the frequently used approach to define frailty, followed by FRAIL (11 studies, 17.2%), TFI (9 studies, 14.1%), FI (7 studies, 10.9%), EFS (1 study, 1.6%), and VES-13 (1 study, 1.6%). Studies using the TFI were excluded from the meta-analysis because the prevalence of frailty estimated by the TFI was significantly higher than that evaluated by other criteria (*p* < 0.001) (Supplementary Material S2), which could be caused by the different conceptual framework to other definitions. Two studies using EFS or VES-13 was also excluded due to the limited number of studies.

### Study Quality

The studies were scored from 4 to 7 (Supplementary Material S3). Two major methodological problems were observed in most studies: 1) None of the studies appropriately reported the prevalence with confidence intervals; 2) Dropouts in each study, response rates, and reasons for non-response were seldom mentioned. Additionally, some studies did not give a clear mean age nor did they include participants in a random way. Accordingly, a potential selection bias in these studies should be noted; a comprehensive and high-quality survey is necessary for further studies.

### Meta-Analysis of the Prevalence of Frailty and Prefrailty

53 studies, where frailty was defined by FI, FFP or FRAIL, were initially analysed. Three studies were considered potential outliers by sensitivity analysis and were further removed (Supplementary Material S4).

**FIGURE 2:**
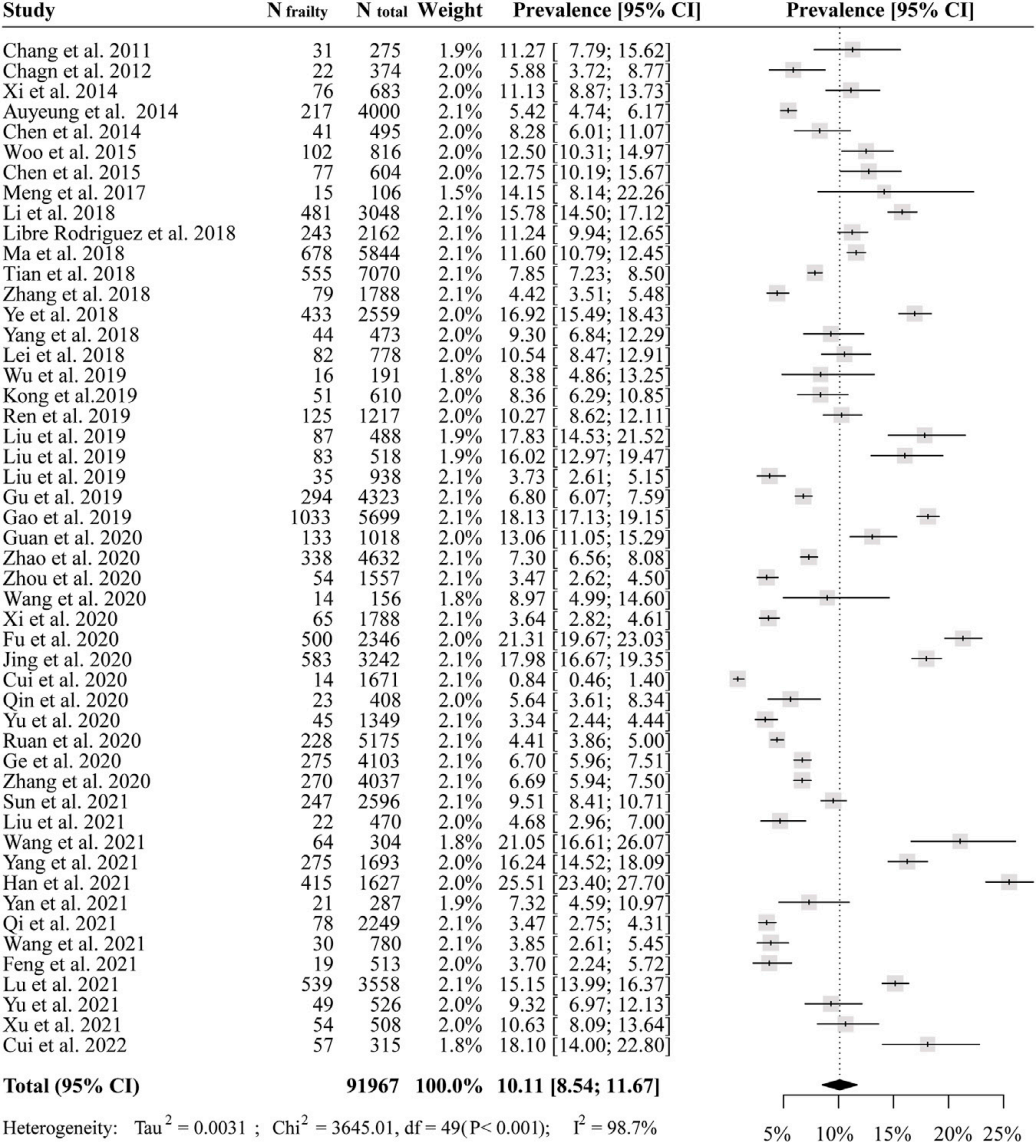
The pooled prevalence of frailty among Chinese community-dwelling older adults (China, 2011–2022).

Finally, this meta-analysis on the prevalence of frailty incorporated 50 studies with 91,967 participants (Figure 2). The pooled prevalence of frailty was 10.1% (95% CI = 8.5–11.6%, I^2^ = 98.7%). 45 studies with 74,020 participants were pooled and the pooled prevalence of prefrailty was 43.9% (95% CI = 40.1–47.8%, I^2^ = 99.5%) (Supplementary Material S5).

### Stratified meta-Analysis for the Prevalence of Frailty and Prefrailty

Table 1 shows the pooled prevalence of frailty stratified by age, sex, urban residence, living arrangement, marriage status, schooling time and geographical region. The pooled prevalence in subjects older than 80 (20.4%) was triple that of participants aged 60–69 (6.2%). Furthermore, the pooled prevalence of frailty was slightly higher in females than in males (10.5% vs. 9.3%, 35 studies), higher in people who lived alone than people who lived with others (11.3% vs. 9.9%, 15 studies), higher in people who had shorter years of schooling (14.0% vs. 8.4%, 25 studies) and lower in married participants than in unmarried ones (13.9% vs. 10.4%, 23 studies).

**TABLE 1:** Pooled prevalence of frailty and prefrailty stratified by diagnosed criteria, age, sex, living arrangement, marriage status, urbanity, schooling time and geographical region (China, 2011–2022).

Community setting	Frailty^a^				Prefrailty^a^			
	N Study	N Participants	Prevalence (95% CI)	I^2^ (%)	N Study	N Participants	Prevalence (95% CI)	I^2^ (%)
Diagnostic criteria								
FFP	33	55,198	9.9 (7.8–11.9)	98.6	29	43,095	47.1 (43.3–50.8)	98.5
FRAIL	11	17,015	10.8 (7.8–13.9)	98.7	11	17,015	37.9 (30.3–45.6)	98.0
FI	8	30,337	10.3 (6.9–13.8)	98.4	5	13,910	38.8 (24.9–29.6)	99.9
TFI	9	7,071	32.4 (24.2–40.7)	97.8	-	-	-	-
Age								
60–69 years	12	16,150	6.2 (3.2–9.2)	98.7	10	10,798	34.9 (26.1–43.6)	98.2
70–79 years	12	10,028	10.2 (6.7–13.7)	97.9	7	3,714	40.0 (29.4–50.6)	97.5
≥80 years	9	2,713	20.4 (15.6–25.2)	88.2	7	1,613	50.9 (38.3–63.6)	92.3
Sex								
Male	35	32,859	9.3 (7.5–11.3)	97.0	31	26,046	46.6 (41.9–51.2)	97.9
Female	35	42,840	10.5 (8.3–12.6)	97.8	31	33,868	45.9 (41.7–50.0)	98.3
Living arrangement								
Alone	15	3,121	11.3 (7.3–15.4)	94.8	14	2,896	50.2 (42.6–57.9)	93.9
Not alone	15	17,091	9.9 (5.6–14.2)	98.3	14	14,720	41.7 (35.1–51.1)	99.3
Marital status								
Married	23	37,897	10.0 (7.5–12.5)	98.7	20	30,654	45.4 (40.4–50.5)	98.8
Unmarried	23	9,646	13.9 (10.4–17.3)	95.5	20	6	49.0 (43.8, 54.3)	97.5
Urbanity								
Rural	6	17,798	14.5 (8.6–20.3)	99.3	5	14,556	49.8 (35.2–64.4)	99.7
Urban	20	23,468	8.7 (6.0–11.0)	98.2	19	20,872	39.0 (32.4–45.6)	99.3
Time of schooling								
<6 years	25	20,932	14.0 (10.7–17.4)	98.0	23	16,903	47.5 (42.2–52.9)	98.2
≥6 years	25	25,748	8.4 (6.2–10.6)	98.0	23	19,818	44.3 (39.4–49.4)	98.6
Geographical region								
Shanghai	10	20,428	7.7 (3.1–12.5)	98.4	10	22,789	40.5 (32.7–48.4)	99.4
Shandong	7	16,390	12.6 (7.3–18.0)	99.0	6	13,148	45.9 (37.6–54.2)	99.2
Beijing	7	10,768	9.4 (6.7–12.2)	95.9	6	8,606	44.7 (28.7–60.6)	99.8
Hong Kong	2	4,816	8.8 (0–12.6)	97.1	3	8,243	47.7 (38.9–56.7)	95.6
Shanxi	2	4,070	9.4 (0–20.6)	99.2	2	4,071	25.8 (0–52.4)	99.6
Anhui	2	3,239	12.3 (5.1–19.6)	91.9	2	2,177	63.6 (54.2–73.0)	85.6
Sichuan	3	1,603	10.43 (8.2–12.7)	54.6	3	1,603	42.1 (23.6–60.6)	98.5
Jiangsu	2	1,944	5.8 (0.7–11.0)	80.9	2	3,237	35.0 (24.6–45.4)	85.3
Taiwan	3	1,114	8.2 (5.3–11.1)	66.4	3	1,114	55.6 (45.5–65.8)	92.8

^a^ Studies that used the TFI as a diagnostic criterion were excluded. I^2^, residual heterogeneity/unaccounted variability. FFP, Fried Frailty Phenotype; FRAIL, the 5-term FRAIL scale; TFI, the Tilburg Frailty Indicator; FI, Rockwood’s Frailty Index; EFS, Edmonton Frailty Scale; VES-13, Vulnerable Elders Survey.

A tremendous regional disparity was observed among nine provinces, with the prevalence ranging from 5.8% in Jiangsu to 12.3% in Anhui province. The prevalence of rural community dwellers (14.5%) was almost two times that of urban community dwellers (8.5%). In addition, cities with higher GDP showed a significantly lower prevalence of frailty than those with lower GDP in the regression analysis (Supplementary Materials S6, S7). Accordingly, good economic conditions were associated with a lower prevalence of frailty.

A similar trend of the prefrailty was observed for factors including age, living arrangement, marital status, urbanity, and schooling time.

### The Risk Factors of Frailty

The odds of being frail for participants aged 70–79 years and 80–89 years was nearly twice (13 studies: OR = 1.81, 95% CI = 1.43–2.29, I^2^ = 84.6%) and four times higher (15 studies: OR = 4.26, 95% CI = 3.19–5.70, I^2^ = 88.9%) than those aged 60–69 years (Table 2).

**TABLE 2:** Associations of frailty with age, sex, educational level, living arrangement and marital status (China, 2011–2022).

	Number of studies	Number of participants	Odds ratio (95% CI)	Heterogeneity (I^2^, %)
Age				
70–79 vs. 60–69^a^years	13	26,451	1.81 (1.43–2.29)	84.6
80+ vs. 60–69 years	15	21,604	4.26 (3.19–5.70)	88.9
Sex				
Male vs. Female^a^	35	75,699	0.89 (0.77–1.01)	85.3
Schooling time				
>6 years vs. ≤6 years^a^	25	46,671	0.60 (0.51–0.72)	81.7
Living arrangement				
Alone vs. Not alone^a^	15	20,212	1.38 (0.99–2.12)	88.3
Marital status				
Married vs. Unmarried^a^	23	47,543	0.64 (0.53–0.77)	84.5

^a^ Taken as the reference group.

Men had an 11% reduction in the odds of being frail compared to women (35 studies: OR = 0.89, 95% CI = 0.77–1.01, I^2^ = 84.6%). People with longer years of schooling time had 37% lower odds of being frail than those who with six or fewer years of education (25 studies: OR = 0.60, 95% CI = 0.52–0.75, I^2^ = 81.7%) (Table 2).

Twenty-nine studies reported marital status and nineteen studies reported living arrangements among different frailty groups. Globally, married elderly were 36% less likely to be frail than unmarried ones (OR = 0.64, 95% CI = 0.53–0.77, I^2^ = 84.5%), and people who lived alone were 38% more likely to be frail than people who did not live alone (OR = 1.39, 95% CI = 0.99–2.12, I^2^ = 88.3%) (Table 2). Similar results were found in the sensitivity analysis for all the risk factors (Supplementary Materials S8–S10).

### Sensitivity Analysis, Meta-regression, and Publication Bias

High heterogeneity was found for the prevalence of frailty (all I^2^ > 75%) was found in the overall analysis and the subgroup analysis; it remained substantial after removing the outliers in the sensitivity analysis (Supplementary Material S4). Meta-regression was applied to examine the influence of age, sex, marital status, live alone, urbanity, sample size, study language, and GDP on the prevalence of frailty (Supplementary Material S11). In the models, age, urbanity and GDP were significant predictors of the prevalence of frailty (all *p* < 0.05) and could explain 11.4%, 10.3%, and 23.3% of the variance, respectively. The influence of age remained moderately significant after adjustment for GDP and urbanity in a multivariate model (*p* = 0.09, R^2^ = 12.4%); A one-year’s increase in age predicted a 1.0% rise in the prevalence of frailty. No evidence of publication bias was observed based on funnel plots and Egger tests (*P* for frailty prevalence = 0.65, *P* for prefrailty prevalence = 0.79) (Supplementary Material S12).

## Discussion

This systematic review and meta-analysis is the most comprehensive assessment of the prevalence of frailty in older Chinese adults living in the community. Twenty-four of 34 Chinese provinces or main areas were included in this study, and nine factors related to frailty, including diagnostic criteria, age, sex, living arrangement, marital status, years of schooling, urbanity, geographical region and economic conditions, were investigated. The results provide the best available strategic information for national public health priorities, such as addressing differences in the prevalence of frailty across cities with different economic conditions, and focusing more attention on people who live alone and unmarried older persons with higher odds of frailty.

The present study showed a similar pooled prevalence of frailty study (10.1%; 95% CI = 8.5%–11.7%) to that of a previously published meta-analysis (10%; 95% CI = 8%–12%) among older people living in community [5]. However, both study found considerable differences between subgroups, indicating a high heterogeneity of frail status among older adults and the urgency to conducting a comprehensive and nationwide survey.

Among the risk factors, age could be the strongest to determine the heterogeneity of frailty. The organs of older adults undergo degenerative changes with increased age, resulting in an accumulated risk of frailty. In the present study, the influence of age on frailty remained significant in meta-regression even after adjusting for GDP and urbanity (Supplementary Material S11). The prevalence of frailty increased steadily from people in their 60s–80s; the odds of being frail for people over 80 reached four times of those aged 60–69 years. All our results suggest that further works is necessary, including frailty prevention, intervention, and policy development vis-à-vis various age groups.

There is not a gold standard criterion to measure frailty; however, two tools, FFP and FI, were commonly used to screen for frailty among a massive population [23]. Differences in the frailty diagnostic method explained large variations in prevalence in a worldwide meta-analysis [21]. Our study showed that the prevalence diagnosed with TFI was significantly higher than that diagnosed with FI, FFP, and FRAIL. The prevalence of frailty assessed by FI (12.7%) was slightly higher than that evaluated by the FFP (11.3%) and FRAIL (10.4%), although the difference was not statistically significant. This trend was also found in the SHARE study (Survey of Health, and Retirement in Europe) and the Rulas study (Rugao longevity and study), both of which documented that FI-based prevalence was around twice higher than FFP-based prevalence [24, 25]. Theoretically, FFP views frailty as a syndrome, whereas the FI approach views frailty as a spectrum of ageing [26, 27]. Both criteria strongly predicted adverse outcomes among community-dwelling older adults [13, 28]. However, the accuracy of these criteria was slightly low [29], and which criterion should be used as the gold standard for frailty screening among community-dwelling populations is still under debate. In the present systematic review, we observed that the FFP was the most used tool in frailty-related investigations for the older population, followed by the FRAIL criteria. Both tools are inexpensive and not time-consuming, thereby making them suitable for large-scale population samples and developing areas.

Underdeveloped regions, especially rural areas, showed a high prevalence of frailty in the present review. This trend is similar to a previous report that middle-income countries appeared to have a higher prevalence of frailty than high-income countries [21]. Economic conditions might be another key risk factor for the prevalence of frailty. Compared to developing areas, highly developed cities generally have more health insurance coverage, longer years of schooling, more educational resources and richer nutrient supplies to prevent disease [30, 31], which were significantly associated with a lower prevalence of frailty [32–34]. Since 2009, great advances have been made in achieving equal access to medical services and insurance coverage across regions of China [6]. Urbanisation has also occurred rapidly in China in recent years, resulting in improved nutritional and dietary patterns. However, large gaps remained in the prevalence of frailty between developed and developing areas according to this systematic review based on studies from 2011 to 2022. In previous studies, the effectiveness of interventions to prevent frailty progression has generally been investigated in older adults. However, economic analysis for these interventions has rarely been performed [35]. Selecting a cost-saving method suitable for underdeveloped areas is necessary for estimating the prevalence and frailty intervention. In addition, education on the primary prevention of frailty should be enhanced in undeveloped areas, as we observed strong associations between frailty and years of schooling time.

Living alone or unmarried were two characteristics significantly correlated with frailty in global meta-analyses [10, 11]. The present meta-analysis showed a similar result for older Chinese adults, among whom the associations between living arrangement, marital status, and frailty have not been systematically estimated. Little evidence exists to explain why unmarried adults and those living alone are more likely to be frail than married adults and adults who live with companions. One potential mechanism is linked to the effects of marriage selection, where healthier individuals are more likely to marry and to stay married [36]. Another mechanism might be that seniors living alone or unmarried have fewer social networks with family and friends, which play an important role in health promotion. They are at risk of social isolation, which is associated with depression, cardiovascular disease, cognitive decline, and increased mortality risk [37–39]. Similar results occurred among unmarried individuals, who are more sensitive to social network than married individuals [40]. Those who lose their partners may experience emotional stress, suffer from changes, and even lose social networks. Likewise, living alone is a kind of social frailty that is a risk factor for physical frailty [41]. Among the studies in this meta-analysis, some confounders including unmarried status (divorced, widowed, never married), could not be adjusted due to design limitations. However, it is highly possible that poor social networks partly contributed to the frailty of Chinese lonely and unmarried older people.

There are several strengths of our study. First, the study followed the protocol according to the PRISMA statements, with a robust search strategy and comprehensive search words using multiple databases. Second, we included the largest number of studies including almost 70% of China’s provinces and regions, and nine frailty-related factors were investigated by pooled analysis. Third, this is the first study to depict a strong association between the increased frailty prevalence and low economic development in different geographical regions. These findings highlighted how to further surveillance-conducting and may have important implications for policy-making on promoting the balanced development of China’s health services among different regions.

Some limitations of this study should be noted. First, few studies were conducted in undeveloped regions or rural areas. Since they had a higher prevalence of frailty than developed regions, the overall frailty prevalence in China could be underestimated. Second, the reviewed studies showed very high levels of heterogeneity in the prevalence of frailty, which was not greatly improved or explained by sensitivity and meta-regression analysis. Frailty reflects highly complex physiological, psychological, and social problems among older adults and varies across individuals. In addition, the different concepts of frailty definition may affect the overall accuracy of prevalence, although nonsignificant differences were observed between FFP, FRAIL, and FI. Thus, it is unlikely that any review can account for all sources of heterogeneity. Third, the sampling strategies for each study were inconsistency. Some samples may not represent the older adults in the whole city, therefore, influencing the generalisability of our results.

In conclusion, although many study on frailty have been performed in China, great disparities in its prevalence were found due to the different definitions of frailty, age groups, geographic regions, and other factors. Seniors who are the oldest, women, unmarried people, have few years of schooling, and live in under-developed areas are at high odds of being frail. The Chinese government should, therefore, pay more attention to these people during its policy-making process.
