# Supplementary material for: Prevalence of Frailty Among Chinese Community-Dwelling Older Adults: A Systematic Review and Meta-Analysis
Source: Int J Public Health. 2023 Aug 1;68:1605964. doi: 10.3389/ijph.2023.1605964 (PMC10425593; doi:10.3389/ijph.2023.1605964)
Supplement: Supplementary file 1 [file DataSheet1.DOCX]

**Supplementary Materials**

**Supplementary material 1** Characteristics and references of included studies

| **Author/Year^a^** | **Province** | **City^b^** | **N^c^** | **Time^d^** | **Comor**  **bidity** | **Sampling^e^** | **Age** | **Female**  **（%）** | **Urbanity** | **Diagnostic** | **Language** | **Setting** | **Prefrail** | **Frail** |
| --- | --- | --- | --- | --- | --- | --- | --- | --- | --- | --- | --- | --- | --- | --- |
| Wang et al. 2021^1^ | Anhui | Bengbu | 400 | 2020 | NA | CS | 69.4 | 57.0 | Urban | TFI | Chinese | Community | - | 14.8 |
| Wu et al. 2019^2^ | Anhui | Hefei* | 191 | 2016-2017 | NA | CS | 71.4 | 48.2 | Urban | FRAIL | Chinese | Community | 58.1 | 8.4 |
| Zhu et al. 2020^3^ | Anhui | Bengbu | 672 | 2019 | NA | RS | 60+ | 58.2 | Urban | TFI | Chinese | Community | 36.2 | 17.7 |
| Li et al. 2018^4^ | Anhui | Maanshan | 3048 | 2016 | NA | CS | 71.6 | 58.5 | Rural | FI | English | Community | 67.8 | 15.8 |
| Shan et al. 2021^5^ | Beijing | Beijing* | 562 | 2019 | NA | CS | 85 | 66.6 | Urban | EFS | Chinese | Community | - | 55.9 |
| Guan et al. 2020^6^ | Beijing | Beijing* | 1018 | 2018-2019 | NA | RS | 69 | 54.0 | Both | FI | Chinese | Community | 50.6 | 13.1 |
| Kong et al.2019^7^ | Beijing | Beijing* | 610 | 2015-2016 | NA | RS | 79.9 | 51.9 | Urban | FRAIL | Chinese | Community | 39.7 | 8.4 |
| Meng et al. 2017^8^ | Beijing | Beijing* | 106 | 2015 | NA | CS | 79.5 | 4.7 | - | FFP | Chinese | Community | 61.3 | 14.2 |
| Zhao et al. 2020^9^ | Beijing | Beijing* | 4632 | 2013-2014 | NA | SRS | 75.4 | 61.2 | - | FFP | English | Community | 62.8 | 7.3 |
| Xi et al. 2014^10^ | Beijing | Beijing* | 683 | 2013 | NA | CS | 74.1 | 82.9 | Urban | FFP | Chinese | Community | 45.7 | 11.1 |
| Zhou et al. 2020^11^ | Beijing | Beijing* | 1557 | 2015-2016 | NA | TP | 74.5 | 58.8 | Urban | FI | Chinese | Community | 8.8 | 3.5 |
| Libre Rodriguez^12^ 2018 | Beijing | Beijing* | 2162 | NA | NA | RS | 73.2 | 56.3 | Both | FFP | English | Community | - | 11.2 |
| Cai et al. 2021^13^ | Guangdong | Guangzhou* | 544 | 2018-2019 | NA | SRS | 70.5 | 65.4 | - | TFI | Chinese | Community | - | 44.9 |
| Ren et al. 2019^14^ | Guangdong | Guangzhou* | 1217 | 2017-2018 | NA | CS | 72 | 55.0 | Urban | FI | Chinese | Community | 30.7 | 10.3 |
| Zhou et al. 2020^15^ | Guizhou | Qinnan | 1849 | 2018-2019 | NA | RS | 71.7 | 56.7 | Rural | TFI | Chinese | Community | - | 26.9 |
| Li et al. 2021^16^ | Hainan | Haikou* | 600 | 2019 | Yes | RS | 60+ | 56.8 | Urban | FFP | Chinese | Community | - | 35.8 |
| Li et al. 2018^17^ | Heilongjiang | Haerbin* | 808 | 2016 | NA | CS | 75.2 | 59.2 | Urban | TFI | Chinese | Community | - | 49 |
| Sun et al. 2021^18^ | Henan | Zhengzhou* | 2596 | 2020 | NA | CS | 60+ | 46.4 | Both | FFP | Chinese | Community | - | 9.5 |
| Auyeung et al. 2014^19^ | HongKong | HongKong* | 4000 | 2001-2003 | NA | RS | 72.48 | 50.0 | Rural | FFP | English | Community | 21.8 | 2.9 |
| Woo et al. 2015^20^ | HongKong | HongKong* | 816 | NA | NA | SRS | 65+ | 85.4 | - | FRAIL | English | Community | 52.5 | 12.5 |
| Liu et al. 2021^21^ | Hubei | Wuhan* | 470 | 2020 | NA | CS | 65+ | 58.1 | Urban | FFP | English | Community | 25.3 | 4.7 |
| Hu et al.2019 ^22^ | Hunan | Shaoyang | 101 | 2018 | Yes | CS | 70.3 | 53.5 | Rural | VES | Chinese | Community | - | 38.6 |
| Wang et al. 2020^23^ | Jiangsu | Suzhou | 1788 | NA | NA | CS | 75.3 | 53.9 | Rural | FFP | Chinese | Community | 30.4 | 3.6 |
| Xi et al. 2020^24^ | Jiangsu | Rugao | 156 | 2014 | Yes | RS | 71.6 | 59.0 | Urban | FRAIL | Chinese | Community | 41 | 9 |
| Qin et al. 2021^25^ | Jilin | Changchun* | 195 | 2018 | Yes | RS | 71.6 | 41.8 | Urban | TFI | English | Community | - | 48.7 |
| Wang et al. 2021^26^ | Jilin | Changchun* | 304 | 2017 | NA | CS | 70.5 | 45.4 | Rural | FFP | English | Community | 50.7 | 21.1 |
| Ma et al. 2018^27^ | National | National | 5844 | 2011-2012 | NA | RS | 60+ | 56.7 | - | FI | English | Community | - | 11.6 |
| Song et al. 2021^28^ | National | National | 6595 | 2017-2018 | NA | RS | 91 | 56.1 | - | FI | English | Community | 59.2 | 35.3 |
| Yang et al. 2021^29^ | Shaanxi | Xi`an* | 1693 | 2018-2019 | NA | TP | 74.6 | 57.5 | Urban | FFP | Chinese | Community | 41 | 16.2 |
| Cui et al.2021^30^ | Shandong | Jinan* | 1091 | 2017 | NA | RS | 70.4 | 41.8 | Both | TFI | Chinese | Community | - | 34.2 |
| Liu et al. 2019^31^ | Shandong | Taian* | 488 | 2018 | Yes | CS | 74.7 | 47.3 | Urban | FFP | Chinese | Community | 39.3 | 17.8 |
| Liu et al. 2019^32^ | Shandong | Taian | 518 | 2017 | NA | RS | 72.1 | 61.4 | Urban | FFP | Chinese | Community | 41.1 | 16 |
| Liu et al. 2019^33^ | Shandong | Jinan* | 938 | 2018 | NA | SRS | 70.5 | 69.0 | Urban | FFP | Chinese | Community | 51 | 3.7 |
| Fu et al. 2020^34^ | Shandong | - | 2346 | 2019 | NA | MSCS | 70.1 | 65.0 | Rural | FFP | English | Community | 64.1 | 21.3 |
| Jing et al. 2020^35^ | Shandong | - | 3242 | 2019 | Yes | MSCS | 70.1 | 63.5 | Rural | FFP | English | Community | - | 18 |
| Tian et al. 2018^36^ | Shandong | - | 1788 | 2015-2016 | Yes | MSS | 69.1 | 66.9 | Urban | FFP | English | Community | 43.5 | 4.4 |
| Zhang et al. 2018^37^ | Shandong | Jinan* | 7070 | 2017 | Yes | SRS | 60+ | 59.7 | Both | FI | English | Community | 36.1 | 7.9 |
| Cui et al. 2020^38^ | Shanghai | Shanghai* | 1671 | 2018 | Yes | TP | 72.3 | 64.9 | Urban | FFP | Chinese | Community | 35.1 | 0.8 |
| Han et al. 2021^39^ | Shanghai | Shanghai* | 1627 | 2018 | NA | RS | 81.6 | 57.7 | Urban | FFP | Chinese | Community | 55.7 | 25.5 |
| Qin et al. 2020^40^ | Shanghai | Shanghai* | 408 | 2018 | NA | RS | 65+ | 41.8 | Urban | FFP | Chinese | Community | 49.3 | 5.6 |
| Xu et al. 2019^41^ | Shanghai | Shanghai* | 500 | 2017-2018 | NA | CS | 76 | 45.4 | Urban | TFI | Chinese | Community | - | 26.2 |
| Yan et al. 2021^42^ | Shanghai | Shanghai* | 287 | 2019 | NA | RS | 70.7 | 34.5 | Urban | FFP | Chinese | Community | 64.1 | 7.3 |
| Yu et al. 2020^43^ | Shanghai | Shanghai* | 1349 | 2018-2019 | NA | RS | 71 | 66.3 | Urban | FFP | Chinese | Community | 36.5 | 3.3 |
| Gu et al. 2019^44^ | Shanghai | Shanghai* | 4323 | 2016 | NA | RS | 70.2 | 58.5 | Rural | FFP | English | Community | 49.4 | 6.8 |
| Qi et al. 2021^45^ | Shanghai | Shanghai* | 2249 | 2018-2019 | NA | RS | 60+ | 54.0 | Urban | FFP | English | Community | 32.9 | 3.5 |
| Ruan et al. 2020^46^ | Shanghai | Shanghai* | 5175 | 2018-2019 | NA | CS | 60+ | 51.9 | Urban | FRAIL | English | Community | 35.9 | 4.4 |
| Wang et al. 2021^47^ | Shanghai | Shanghai* | 780 | 2019 | NA | CS | 66.9 | 57.1 | Urban | FFP | English | Community | 47.7 | 3.8 |
| Ye et al. 2018^47^ | Shanghai | Shanghai* | 2559 | 2017 | NA | MRS | 66.1 | 57.4 | Urban | FRAIL | English | Community | 39.5 | 16.9 |
| Gao et al. 2019^49^ | Shanghai, Henan | - | 5699 | 2017-2018 | NA | RS | 66.4 | 50.7 | Urban | FRAIL | English | Community | 38.3 | 18.1 |
| Feng et al. 2021^50^ | Shanxi | Taiyuan* | 513 | 2018-2019 | Yes | RS | 72.6 | 63.7 | Urban | FRAIL | Chinese | Community | 12.3 | 3.7 |
| Wang et al. 2021^51^ | Shanxi | Pingyao | 483 | 2020 | NA | TP | 73.3 | 46.8 | Rural | FFP | Chinese | Community | - | 42.7 |
| Lu et al. 2021^52^ | Shanxi | - | 3558 | 2019 | Yes | MRCS | 69.9 | 48.9 | Rural | FFP | English | Community | 39.4 | 15.1 |
| Yang et al. 2018^53^ | Sichuan | Dujiangyan | 473 | 2014 | NA | TP | 72.6 | 60.5 | Urban | FRAIL | Chinese | Community | 24.3 | 9.3 |
| Yu et al. 2021^54^ | Sichuan | Chengdu* | 526 | 2017-2018 | Yes | RS | 72.9 | 58.0 | Urban | FFP | Chinese | Community | 45.4 | 9.3 |
| Chen et al. 2015^55^ | Sichuan | Chengdu,Suijing | 604 | 2010-2012 | Yes | CS | 70.6 | 42.1 | - | FFP | English | Community | 56.5 | 12.7 |
| Li et al. 2019^56^ | Sichuan | Chengdu, et al. | 459 | 2017-2018 | Yes | SRS | 74.1 | 56.9 | Both | TFI | Chinese | Community | - | 30.7 |
| Ge et al. 2020^57^ | Sichuan, et al. | - | 4103 | 2018 | Yes | RS | 67.8 | 58.3 | - | FFP | English | Community | - | 6.7 |
| Chen et al. 2014^58^ | Taiwan | - | 495 | NA | NA | SRS | 73.4 | 48.3 | Urban | FFP | English | Community | 45.9 | 8.3 |
| Chang et al. 2011^59^ | Taiwan | - | 275 | NA | NA | RS | 71.1 | 53.8 | Urban | FFP | English | Community | 58.5 | 11.3 |
| Chang et al. 2012^60^ | Taiwan | - | 374 | 2011 | Yes | CS | 74.6 | 52.7 | Urban | FFP | English | Community | 62.8 | 5.9 |
| Lei et al. 2018^61^ | Tianjin | Tianjin* | 778 | 2016 | NA | RS | 60+ | 62.1 | Urban | FFP | Chinese | Community | 42.9 | 10.5 |
| Zhang et al. 2020^62^ | Yunan, et al. | - | 4037 | 2018 | Yes | MRCS | 67.8 | 58.2 | - | FFP | English | Community | 47 | 6.7 |
| Cui et al. 2022^63^ | Xinjiang | Shihezi | 315 | 2020-2021 | NA | RS | 72.1 | 56.9 | Urban | FRAIL | Chinese | Community | 28.9 | 18.1 |
| Xu et al. 2021^64^ | Zhejiang | Lishui | 508 | 2018 | NA | CS | 70.1 | 55.5 | Urban | FRAIL | Chinese | Community | 48.4 | 10.6 |

FFP, Fried Frailty Phenotype; FRAIL, the 5-term FRAIL scale; TFI, Tilburg Frailty Indicator; FI, the Rockwood’s Frailty Index; EFS, Edmonton Frailty Scale; VES-13, Vulnerable Elders Survey

^a^ Additional reference

^b^ Capital cities of the provinces

^c^ Number of the participants.

^d^ The time when studies were performed.

^e^ The method of sampling; CS, convenience sampling; RS, random sampling; SRT, stratified random sampling; MRCS, multi state cluster sampling; TP, total population;

**Additional References***

1. Wang C, Xie H, Cai W. Analysis on the frailty of the elderly in community and its influencing factor. Chinese Journal of General Practice 2021;9(4):625-627. doi:10.16766 /j.cnki. issn.1674－4152.001878. (In Chinese)
2. Wu M, Yu W, Ge Q, Xu K, Wu K. Research on gait characteristics under dual-task walking among the community elderly with different frailty states. Journal of Nursing Science. doi:10.3870/j.issn.1001-4152.2019.01.016. (In Chinese)
3. Zhu Y, Zhang L, Xie H, Sun T, Su X, Feng J. Analysis of the current situation of frailty among the elderly in the community and the correlation between cardiovascular function and frailty. Chinese General Practice Nursing 2020; 18(19):2318-2322. doi:10.12104/j.issn.1674-4748.2020.19.004. (In Chinese)
4. Li J, Zhao D, Dong B, Yu D, Ren Q, Chen J, Qin Q, Bi P, Sun Y: Frailty index and its associations with self-neglect, social support and sociodemographic characteristics among older adults in rural China. Geriatr Gerontol Int 2018, 18(7):987-99
5. Shan Q, Hu B, Chen M. Correlation of potentially inappropriate medication and frailty in community-dwelling elderly. Chin J Mult Organ Dis Elderly; 20(08):581-585. doi:10.11915/j.issn.1671-5403.2021.08.121. (In Chinese)
6. Guan X, Wang D, Qiao L, et al. Investigation of current condition of frailty of 1 018 elderly residents in Beijing and analysis of TCM syndromes. CJTCMP 2020; 35(10):5210-5213. doi:NA. (In Chinese)
7. Kong J, Zhang J, Fan X, Cui Y. Application of two frailty assessment tools in the screening of frail elderly persons in the community. Chin J Clin Healthc 2019; 22(05):604-608. doi:10.3969/J.issn.1672-6790.2019.05.008. (In Chinese)
8. Meng L, Shi J, Zhou B, et al. Values of frailty phenotype and frailty index in assessment of frailty for Chinese elderly. Chin J Mult Organ Dis Elderly 2017; 16(05):321-325. doi:10.11915/j.issn.1671-5043.2017.05.075. (In Chinese)
9. Zhao J, Chhetri JK, Ji S, Ma L, Dan X, Chan P: Poor self-perceived health is associated with frailty and prefrailty in urban living older adults: A cross-sectional analysis. Geriatr Nur *(New York, NY)* 2020; 41(6):754-760.
10. Xi X, Guo G. The current status and influencing factors of frailty among elders in community. Community Health Nursing 2014; 14(12):1315-1319. doi:10.3969/.issn.1672-1756.201412.024. (In Chinese)
11. Zhou B, Tao Y, Shi J, Yu P. Analysis fo the frailty status and its related factors among elderly adults of urban community in Beijing. Chin J Geriatr 2020; 30(2):214-219. doi:10.3760/cma.j.issn.0254-9026.2020.02.021. (In Chinese)
12. Llibre Rodriguez JJ, Prina AM, Acosta D, Guerra M, Huang Y, Jacob KS, Jimenez-Velasquez IZ, Salas A, Sosa AL, Williams JD *et al*: The Prevalence and Correlates of Frailty in Urban and Rural Populations in Latin America, China, and India: A 10/66 Population-Based Survey. J Am Med Dir Assoc 2018, 19(4):287-295.e284.
13. Cai S, Wei L, Peng X, et al. The frailty, social support and self-perceived burden of the elderly in the community in Guangzhou. Chinese Jorunal of Gerontology 2021; 41(07):1520-1524. doi:10.3969/j.issn.1005-9202.2021.07.049. (In Chinese)
14. Ren Q, Zhou J, Zheng Y, et al. Prevalence and influencing factors of frailty among elderly people in Guangzhou city. Chin J Public Health 2019; 35(04):413-417. (In Chinese)
15. Zhou Q, She F, Yang J, et al. Investigate frailty status and analysis its influencing factors of the elderly in Qiannan rural minority arears of Guizhou. Chin J Dis Control Prev 2020; 24(10):1139-1143. doi:10.16462/j.cnki.zhjbkz.2020.10.006. (In Chinese)
16. Li X, Mao Y, Pan A, et al. Frailty of the elderly in the communities of Haikou municipality and its influencing factors. Chin J Mult Organ Dis Elderly 2021; 20(11):829-833. Doi:10.11915/j.issn.1671-5403.2021.11.173. (In Chinese)
17. Li Y, Zhang L, Luo Y. Survey of status and influential factors of elderly frailty in community in Harbin city. Chinese Nursing Research 2018; 32(02):224-228. doi:10.3969/j.issn.1009-6493.2018.02.016. (In Chinese)
18. Sun H, Qiao Y, Xia Y, Lu Z, Wang Z, Zhou L. Frailty and cognitive frailty of the elderly in Zhengzhou community. South China J Prev Med 2021; 47(10):1262-1266. do Su i:10.12183/j.scjpm.2021.1262. (In Chinese)
19. Auyeung TW, Lee JS, Leung J, Kwok T, Woo J: The selection of a screening test for frailty identification in community-dwelling older adults. J Nutr Health Aging 2014, 18(2):199-203.
20. Woo J, Yu R, Wong M, Yeung F, Wong M, Lum C: Frailty Screening in the Community Using the FRAIL Scale. J Am Med Dir Assoc 2015, 16(5):412-419.
21. Liu A, Peng Y, Zhu W, Zhang Y, Ge S, Zhou Y, Zhang K, Wang Z, He P: Analysis of Factors Associated With Depression in Community-Dwelling Older Adults in Wuhan, China. Front Aging Neurosci 2021, 13:743193.
22. Hu Y, Liu Y, Li H, Zou Y, Shi Z. Status and influencing factors fo frailty among elderly people aged 60 or above in a remote area in Hu’nan province. Chin J Mod Nus 2019; 25(17):2133-2138. doi:10.3760/cma.j.issn.1674-2907.2019.17.006. (In Chinese)
23. Xi J, Yuan H, Wang L, Huang L. Correlation between frailty and sleep quality among the elderly in the community. Chinese Journal of Gerotology 2020; 40(20):4461-4464. doi:10.3969/j.issn.1005-9202.2020.20.060. (In Chinese)
24. Wang X, Pei Y. Adverse Outcomes of Frailty among the Elderly in China: the RuLAS Study. Population & Development 2020; 26(04):43-50. doi:NA. (In Chinese)
25. Qin Y, Li J, McPhillips M, Lukkahatai N, Yu F, Li K: Association of fear of falling with frailty in community-dwelling older adults: A cross-sectional study. Nurs Health Sci 2021, 23(2):516-524.
26. Wang S, Zhao M, Shi Y, Zhang M, Ying J, Li H, Li Y, Xing Z, Zhang H, Sun J: Associations of frailty, loneliness and the quality of life of empty nesters: A cross-sectional study in rural areas. Int J Nurs Pract 2021:e12947.
27. Ma L, Tang Z, Zhang L, Sun F, Li Y, Chan P: Prevalence of Frailty and Associated Factors in the Community-Dwelling Population of China. J Am Geriatr Soc 2018, 66(3):559-564.
28. Song Y, Deng Y, Li J, Hao B, Cai Y, Chen J, Shi H, Xu W: Associations of falls and severe falls with blood pressure and frailty among Chinese community-dwelling oldest olds: The Chinese Longitudinal Health and Longevity Study. Aging 2021, 13(12):16527-16540.
29. Yang J, Wang A, Shang L, et al. Dietary pattern and its correlation with frailty in the elderly from a community in Xi'an. Chin J Mult Organ Dis Elderly 2021; 20(10):738-744. doi:10.11915/j.issn.1671-5403.2021.10.154. (In Chinese)
30. Cui G, Li S, Yin Y, Chen L, Liu X, Yu P. Association of Frailty with Sleep Quality and TCM-based Constitution among Elderly People. Chinese General Practice 2021; 24(09):1082-1087. doi:10.12114/j.issn.1007-9572.2020.00.620. (In Chinese)
31. Liu X, Liu T, Yu W, Chen H, Zhang A. Status of frailty and its effect path on quality of life among the elderly in community. Chinese Nursing Research 2019; 33(21):3645-3649. doi:10. 12102/jissn.1009-6493.2019.21.002. (In Chinese)
32. Liu T, Zhang A, Zhang Y, Wu X. Effects of depression and psychological resilience on self-care ability of the elderly and the moderating effect of frailty. Chinese Journal of Gerotology 2019; 39(02):448-452. doi:10.3969/j.issn.1005-9202.2019.02. 063. (In Chinese)
33. Liu X, Qiao X, Jin Y, Si H, Wang C. The mediating role of malnutrition between depression and frailty in community-based elderly. Chinese Journal of Gerotology 2019; 39(18):4592-4595. doi:10.3969/j.issn.1005-9202.2019.18.068. (In Chinese)
34. Fu P, Zhou C, Meng Q: Associations of Sleep Quality and Frailty among the Older Adults with Chronic Disease in China: The Mediation Effect of Psychological Distress. Int J Environ Res Public Health 2020, 17(14).
35. Jing Z, Li J, Wang Y, Ding L, Tang X, Feng Y, Zhou C: The mediating effect of psychological distress on cognitive function and physical frailty among the elderly: Evidence from rural Shandong, China. J Affect Disord 2020, 268:88-94.
36. Tian X, Wang C, Qiao X, Liu N, Dong L, Butler M, Si H, Jin Y: Association between pain and frailty among Chinese community-dwelling older adults: depression as a mediator and its interaction with pain. *Pain* 2018, 159(2):306-313.
37. Zhang J, Xu L, Sun L, Li J, Qin W: Gender difference in the association of frailty and health care utilization among Chinese older adults: results from a population-based study. Aging Clin Exp Res 2020, 32(10):1985-1991.
38. Cui Y, Zhang Y, Huang Y, et al. A study on the prevalence of frailty of people over 55 years old in a community of Shanghai and its correlation with comorbidities. Geriatr Health Care 2020; 26(04):542-562. doi:NA. (In Chinese)
39. Han J, Wang J, Wang Y, Xie B. Prevalence and associated factors of cognitive impairment and physical frailty among community-dwelling older adults aged 75 years and above. Fudan Univ J Med Sci 2021; 48(04):494-544. doi:10.3969/j.issn.1672-8467.2021.04.011. (In Chinese)
40. Qin L, Liang Z, GE L, Chen L, Huo Y, Li Q. Influencing factors of frailty syndrome in elderly people in the community. Chinese General Practice 2020; 23(05):598-603. doi:10.12114/j.issn.1007-9572.2020.00.060. (In Chinese)
41. Xu CH, Meng CH. Analysis of the incidence and risk factors of senile frailty. Shanxi Med J 2019; 48(06):650-652. doi:10.3969/j.issn.0253-9926.2019.06.003. (In Chinese)
42. Yan H, Chen Y, Wang Y, Chen H, Wang J, Gao J. Association of metabolic syndrome with frailty among older adults in communities. Chin J Prev Contr Chron Dis 2021; 29(05):347-350. doi:10.16386/j.cjpccd.issn.1004-6194.2021.05.006. (In Chinese)
43. Yu J, Zhang Y, Huang Y, et al. Study on the correlation between physical frailty and sarcopenia in the elderly dwelling in communities of Shanghai. Geriatr HeaHh Care 2020; 26(04):519-523. doi:NA. (In Chinese)
44. Gu J, Chen H, Gu X, Sun X, Pan Z, Zhu S, Young D: Frailty and Associated Risk Factors in Elderly People with Health Examination in Rural Areas of China. Iran J Public Health 2019, 48(9):1663-1670.
45. Qi Z, Wu BL, Chen C, Yu ZH, Shen DZ, Chen JL, Zhao HB, Sun L: Symptoms Based on Deficiency Syndrome in Traditional Chinese Medicine Might Be Predictor of Frailty in Elderly Community Dwellers. *Evidence-based complementary and alternative medicine : eCAM* 2021, 2021:9918811.
46. Ruan Q, Xiao F, Gong K, Zhang W, Zhang M, Ruan J, Zhang X, Chen Q, Yu Z: Prevalence of Cognitive Frailty Phenotypes and Associated Factors in a Community-Dwelling Elderly Population. J Nutr Health Aging 2020, 24(2):172-180.
47. Wang Y, Huang Y, Wu H, He G, Li S, Chen B: Association between Dietary Patterns and Frailty Prevalence in Shanghai Suburban Elders: A Cross-Sectional Study. Int J Environ Res Public Health 2021, 18(20).
48. Ye B, Gao J, Fu H: Associations between lifestyle, physical and social environments and frailty among Chinese older people: a multilevel analysis. BMC Geriatr 2018, 18(1):314.
49. Gao J, Jia Y, Dai J, Fu H, Wang Y, Yan H, Zhu Y, Nie X: Association of Fruit and Vegetable Intake and Frailty among Chinese Elders: A Cross-Sectional Study in Three Cities. J Nutr Health Aging 2019, 23(9):890-895.
50. Feng Q, Bian M, Du Y. Prevalence and Influencing Factors of Frailty among Elderly People in the Community. Chinese General Practice 2021; 24(24):3032-3038. doi:10.12114/j.issn.1007-9572.2021.00.530. (In Chinese)
51. Wang Zh, Gao H, Song G. Frailty prevalence and influncing factors among aged in rural areas of Shanxi province. Journal of Nursing Science 2021; 36(05):88-91. doi:10.3870/j.issn.1001-4152.2021.05.088. (In Chinese)
52. Lu J, Guo QQ, Wang Y, Zuo ZX, Li YY: The Evolutionary Stage of Cognitive Frailty and Its Changing Characteristics in Old Adults. J Nutr Health Aging 2021, 25(4):467-478.
53. Yang F, Wang S, Qin H, et al. Effect of frailty syndrome on falls in Chinese elderly diabetics in the communities: a prospective cohort study. Chin J Epidemiol 2018; 39(06):776-780. doi:10.12114/j.issn.1007-9572.2021.00.530. (In Chinese)
54. Yu J, Gao J, Bai D, et al. Status and influencing factors of frailty among the elderly in Chengdu community. Chinese Journal of Gerontology 2021; 41(09):1972-1977. doi:10.3969/j.issn.1005-9202.2021.09.052. (In Chinese)
55. Chen S, Hao Q, Yang M, Yue J, Cao L, Liu G, Zou C, Ding X, Pu H, Dong B: Association between Angiotensin-converting enzyme insertion/deletion polymorphisms and frailty among chinese older people. J Am Med Dir Assoc 2015, 16(5):438.e431-436.
56. Li Y, Xiong M, Zhang Y, Su Y. The current status and influencing factors fo frailty among community-dwelling old adults. Chin J Behav Med & Brain 2019; 28(11):1020-1024. doi:10.3760/j.issn.1672-6554.2019.11.012. (In Chinese)
57. Ge M, Zhang Y, Zhao W, Yue J, Hou L, Xia X, Zhao Y, Liu X, Dong B, Ge N: Prevalence and Its Associated Factors of Physical Frailty and Cognitive Impairment: Findings from the West China Health and Aging Trend Study (WCHAT). J Nutr Health Aging 2020, 24(5):525-533.
58. Chen L: Prevalence and Associated Factors of Frailty Among Elderly People in Taiwan. In*.*: Int J Gerontol; 2014: 114-119.
59. Chang CI, Chan DC, Kuo KN, Hsiung CA, Chen CY: Prevalence and correlates of geriatric frailty in a northern Taiwan community. J Formos Med Assoc 2011, 110(4):247-257.
60. Chang YW, Chen WL, Lin FG, Fang WH, Yen MY, Hsieh CC, Kao TW: Frailty and its impact on health-related quality of life: a cross-sectional study on elder community-dwelling preventive health service users. PloS one 2012, 7(5):e38079.
61. Lei P, Liu Ch, Gao Y, Xue M. Psychosocial Factors and Frailty in Community-dwelling Older People. Chines General Practice 2018; 21(02):180-185. doi:10.3969/j.issn.1007-9572.2018.02.12. (In Chinese)
62. Zhang Y, Ge M, Zhao W, Hou L, Xia X, Liu X, Zuo Z, Zhao Y, Yue J, Dong B: Association Between Number of Teeth, Denture Use and Frailty: Findings from the West China Health and Aging Trend Study. J Nutr Health Aging 2020, 24(4):423-428.
63. Cui Ch, Dong A, Yang Q. Mediating effect of leisure activities on cognitive frailty and depression in community-dwelling elderly. *Chin J Mult Organ Dis Elderly* 2022: 21(01):45-48. doi:10.11915/j.issn.1671-5403.2022.01.010. (In Chinese)
64. Xu L, Zhang L, Ye L, Li C. Risk factors and risk prediction model construction of elderly frailty in community. Chinese Journal of Gerontology 2021; 41(01):170-173. doi:10.3969/j.issn.1005-9202.2021.01.048. (In Chinese)

#For the paper in which there is not an English title and abstract, Titles were translated into English by google translator.

**Supplementary material 2** The prevalence of frailty according to different diagnostic criteria. The differences between frailty prevalence diagnosed with FFP, FI, FRAIL, and TFI criteria were tested by Wilcoxon rank-sum test (All *P* < 0.01). The frailty prevalence was significantly higher according to TFI criteria compared to that diagnosed by FFP, FI, or FRAIL, while non-significance was found among the prevalence among FFP, FI, and FRAIL groups. Accordingly, we removed the TFI-based studies. EFS- and VES-based studies were also removed because there was only one study for each criterion.


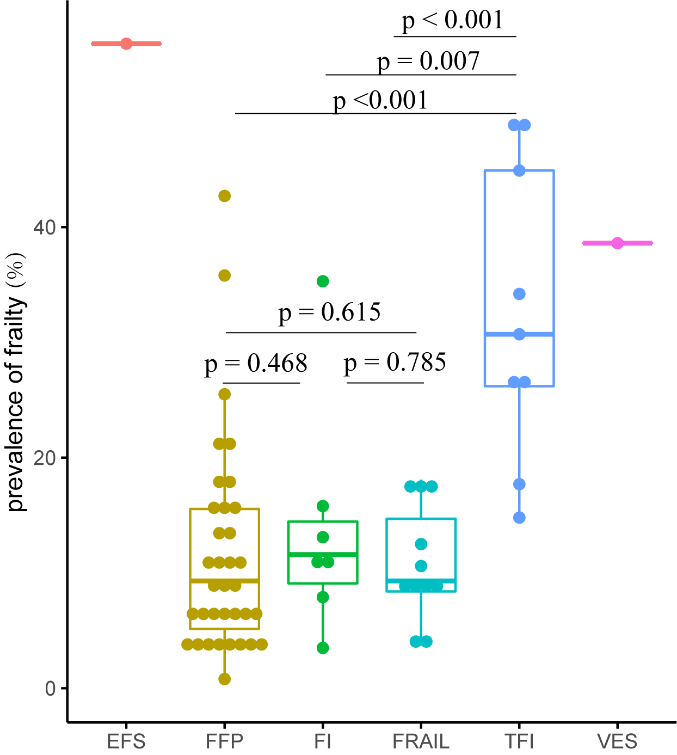


**Supplementary material 3.** JBI critical appraisal checklist for studies reporting prevalence data.

| **Author/Year^#^** | **Q1** | **Q2** | **Q3^a^** | **Q4** | **Q5^b^** | **Q6** | **Q7** | **Q8** | **Q9** | **Total score**  **（Number of “yes”）** |
| --- | --- | --- | --- | --- | --- | --- | --- | --- | --- | --- |
| Wang et al. 2021^1^ | yes | no | yes | yes | yes | yes | yes | no | unclear | 6 |
| Wu et al. 2019^2^ | yes | no | yes | yes | yes | yes | yes | no | unclear | 6 |
| Zhu et al. 2020^3^ | yes | yes | yes | yes | yes | yes | yes | no | unclear | 7 |
| Li et al. 2018^4^ | yes | no | yes | yes | yes | yes | yes | no | unclear | 6 |
| Shan et al. 2021^5^ | yes | no | yes | yes | yes | yes | yes | no | unclear | 6 |
| Guan et al. 2020^6^ | yes | yes | yes | yes | yes | yes | yes | no | unclear | 7 |
| Kong et al.2019^7^ | yes | yes | yes | yes | yes | yes | yes | no | unclear | 7 |
| Meng et al. 2017^8^ | yes | no | yes | yes | yes | yes | yes | no | unclear | 6 |
| Zhao et al. 2020^9^ | yes | yes | yes | yes | yes | yes | yes | no | unclear | 7 |
| Xi et al. 2014^10^ | yes | no | yes | yes | yes | yes | yes | no | unclear | 6 |
| Zhou et al. 2020^11^ | yes | yes | yes | yes | yes | yes | yes | no | unclear | 7 |
| Libre Rodriguez^12^ 2018 | yes | yes | yes | yes | yes | yes | yes | no | unclear | 7 |
| Cai et al. 2021^13^ | yes | yes | yes | yes | yes | yes | yes | no | unclear | 7 |
| Ren et al. 2019^14^ | yes | no | yes | yes | yes | yes | yes | no | unclear | 6 |
| Zhou et al. 2020^15^ | yes | yes | yes | yes | yes | yes | yes | no | unclear | 7 |
| Li et al. 2021^16^ | yes | yes | yes | no | unclear | yes | yes | no | unclear | 5 |
| Li et al. 2018^17^ | yes | no | yes | yes | yes | yes | yes | no | unclear | 6 |
| Sun et al. 2021^18^ | yes | no | yes | no | unclear | yes | yes | no | unclear | 4 |
| Auyeung et al. 2014^19^ | yes | yes | yes | yes | yes | yes | yes | no | unclear | 7 |
| Woo et al. 2015^20^ | yes | yes | yes | no | unclear | yes | yes | no | unclear | 5 |
| Liu et al. 2021^21^ | yes | no | yes | no | unclear | yes | yes | no | unclear | 4 |
| Hu et al.2019 ^22^ | yes | no | yes | yes | yes | yes | yes | no | unclear | 6 |
| Wang et al. 2020^23^ | yes | no | yes | yes | yes | yes | yes | no | unclear | 6 |
| Xi et al. 2020^24^ | yes | yes | yes | yes | yes | yes | yes | no | unclear | 7 |
| Qin et al. 2021^25^ | yes | yes | yes | no | unclear | yes | yes | no | unclear | 5 |
| Wang et al. 2021^26^ | yes | no | yes | yes | yes | yes | yes | no | unclear | 6 |
| Ma et al. 2018^27^ | yes | yes | yes | no | unclear | yes | yes | no | unclear | 5 |
| Song et al. 2021^28^ | yes | yes | yes | yes | no | yes | yes | no | unclear | 6 |
| Yang et al. 2021^29^ | yes | yes | yes | yes | yes | yes | yes | no | unclear | 7 |
| Cui et al.2021^30^ | yes | yes | yes | yes | yes | yes | yes | no | unclear | 7 |
| Liu et al. 2019^31^ | yes | no | yes | yes | yes | yes | yes | no | unclear | 6 |
| Liu et al. 2019^32^ | yes | yes | yes | yes | yes | yes | yes | no | unclear | 7 |
| Liu et al. 2019^33^ | yes | yes | yes | yes | yes | yes | yes | no | unclear | 7 |
| Fu et al. 2020^34^ | yes | yes | yes | yes | yes | yes | yes | no | unclear | 7 |
| Jing et al. 2020^35^ | yes | yes | yes | yes | yes | yes | yes | no | unclear | 7 |
| Tian et al. 2018^36^ | yes | yes | yes | no | no | yes | yes | no | unclear | 5 |
| Zhang et al. 2018^37^ | yes | yes | yes | yes | yes | yes | yes | no | unclear | 7 |
| Cui et al. 2020^38^ | yes | yes | yes | yes | yes | yes | yes | no | unclear | 7 |
| Han et al. 2021^39^ | yes | yes | yes | yes | yes | yes | yes | no | unclear | 7 |
| Qin et al. 2020^40^ | yes | yes | yes | yes | yes | yes | yes | no | unclear | 7 |
| Xu et al. 2019^41^ | yes | no | yes | yes | yes | yes | yes | no | unclear | 6 |
| Yan et al. 2021^42^ | yes | yes | yes | yes | yes | yes | yes | no | unclear | 7 |
| Yu et al. 2020^43^ | yes | yes | yes | yes | yes | yes | yes | no | unclear | 7 |
| Gu et al. 2019^44^ | yes | yes | yes | yes | yes | yes | yes | no | unclear | 7 |
| Qi et al. 2021^45^ | yes | yes | yes | no | unclear | yes | yes | no | unclear | 5 |
| Ruan et al. 2020^46^ | yes | no | yes | no | unclear | yes | yes | no | unclear | 4 |
| Wang et al. 2021^47^ | yes | no | yes | yes | yes | yes | yes | no | unclear | 6 |
| Ye et al. 2018^47^ | yes | Yes | yes | yes | yes | yes | yes | no | unclear | 7 |
| Gao et al. 2019^49^ | yes | yes | yes | yes | yes | yes | yes | no | unclear | 7 |
| Feng et al. 2021^50^ | yes | yes | yes | yes | yes | yes | yes | no | unclear | 7 |
| Wang et al. 2021^51^ | yes | yes | yes | yes | yes | yes | yes | no | unclear | 7 |
| Lu et al. 2021^52^ | yes | yes | yes | yes | yes | yes | yes | no | unclear | 7 |
| Yang et al. 2018^53^ | yes | yes | yes | yes | yes | yes | yes | no | unclear | 7 |
| Yu et al. 2021^54^ | yes | yes | yes | yes | yes | yes | yes | no | unclear | 7 |
| Chen et al. 2015^55^ | yes | no | yes | yes | yes | yes | yes | no | unclear | 6 |
| Li et al. 2019^56^ | yes | yes | yes | yes | yes | yes | yes | no | unclear | 7 |
| Ge et al. 2020^57^ | yes | yes | yes | yes | yes | yes | yes | no | unclear | 7 |
| Chen et al. 2014^58^ | yes | yes | yes | yes | yes | yes | yes | no | unclear | 7 |
| Chang et al. 2011^59^ | yes | yes | yes | yes | yes | yes | yes | no | unclear | 7 |
| Chang et al. 2012^60^ | yes | no | yes | yes | yes | yes | yes | no | unclear | 6 |
| Lei et al. 2018^61^ | yes | yes | yes | no | unclear | yes | yes | no | unclear | 5 |
| Zhang et al. 2020^62^ | yes | yes | yes | yes | yes | yes | yes | no | unclear | 7 |
| Cui et al. 2022^63^ | yes | yes | yes | yes | yes | yes | yes | no | unclear | 7 |
| Xu et al. 2021^64^ | yes | no | yes | yes | yes | yes | yes | no | unclear | 6 |

^a^ The sample size was calculated using the suggested function: n = z2 p (1 - p) / e2 (JBI Critical, z = 1.96 for a confidence level (α) of 90%, p = 10%, e = margin of error of 0.05). A sample size of 99 is adequate in prevalence analysis.

^b^ Those studies with unclear mean age or age ranges of the subjects were marked as unclear.

Q1, was the sample frame appropriate to address the target population?

Q2, were study participants sampled in an appropriate way?

Q3, was the sample size adequate?

Q4, were the study subjects and the setting described in detail?

Q5, was the data analysis conducted with sufficient coverage of the identified sample?

Q6, were valid methods used for the identification of the condition?

Q7, was the condition measured in a standard, reliable way for all participants?

Q8, was there appropriate statistical analysis?

Q9, was the response rate adequate, and if not, was the low response rate managed appropriately?

**Supplementary material 4** Sensitivity analysis for the pooled prevalence of frailty. In sensitivity analysis (leave-one-out analyses), each of the 53 studies which used FFP, FI, or FRAIL as diagnostic criteria was removed in turn one at a time, and the summary proportion was re-estimated based on the remaining 52 studies. Externally standardized residual, differences in fits values, Cook’s distance, covariance ratio, τ² and Q-statistics for heterogeneity were used to investigate the outliners (red points) which could potentially affect the pooled prevalence of frailty. Red points were as outliers.


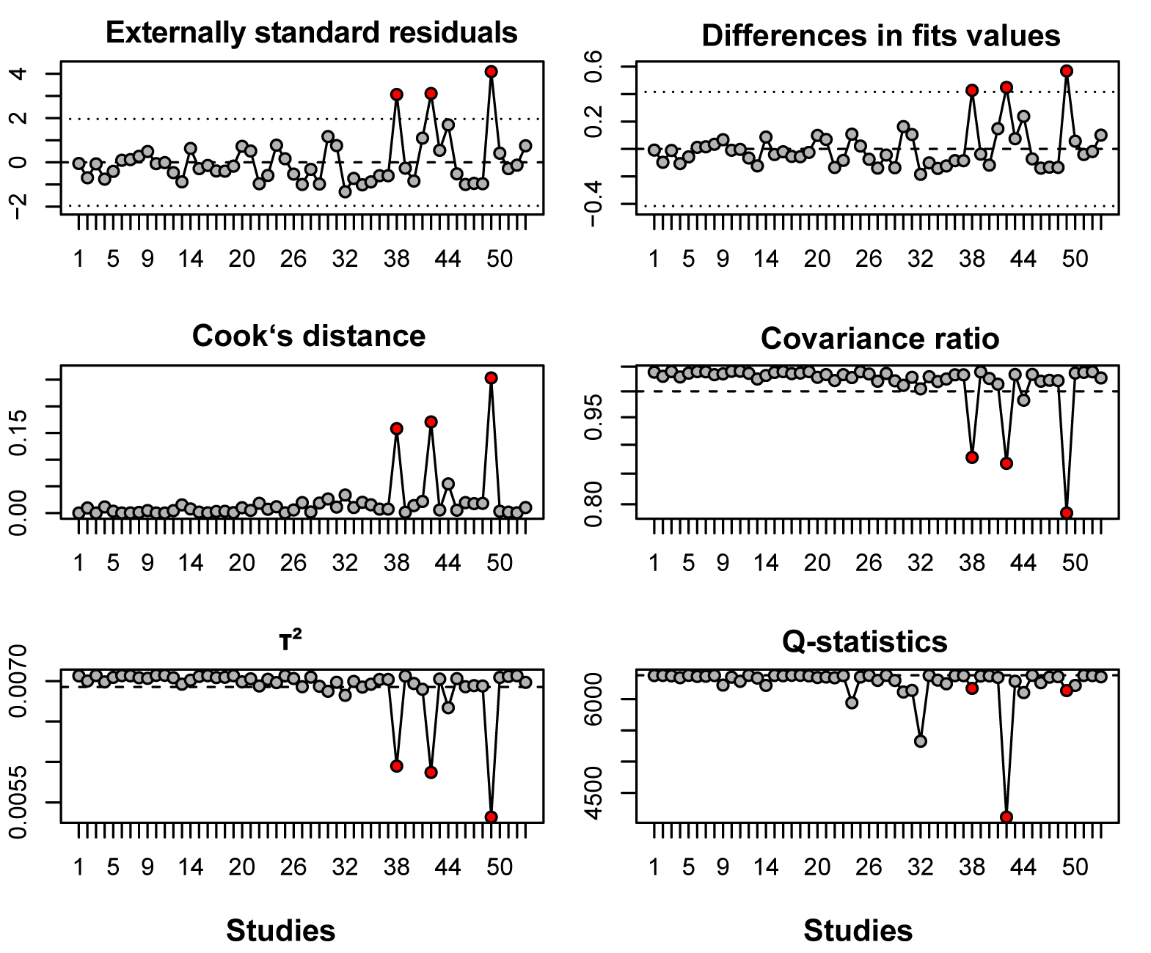


Three outliers (Li X, et al. 2021; Song Y, et al. 2021; Wang Zh, et al. 2021) were identified in the sensitivity analysis. One study (Song Y, et al. 2021) was a national wide investigation, had a sample size of 6595, aged over 90 years, and diagnosed by FI. The older age of this population might be the reason why it had significantly higher frailty prevalence. The significant higher prevalence of frailty in the two studies might come from a potential selection bias. Although we are not very certain about their protentional selection bias, we choose to removed them in the meta-analysis of frailty and prefrailty prevalence due to the unacceptable high value.

**Supplementary material 5** Random-effect pooled prevalence of prefrailty among the Chinese community-dwelling older adults.


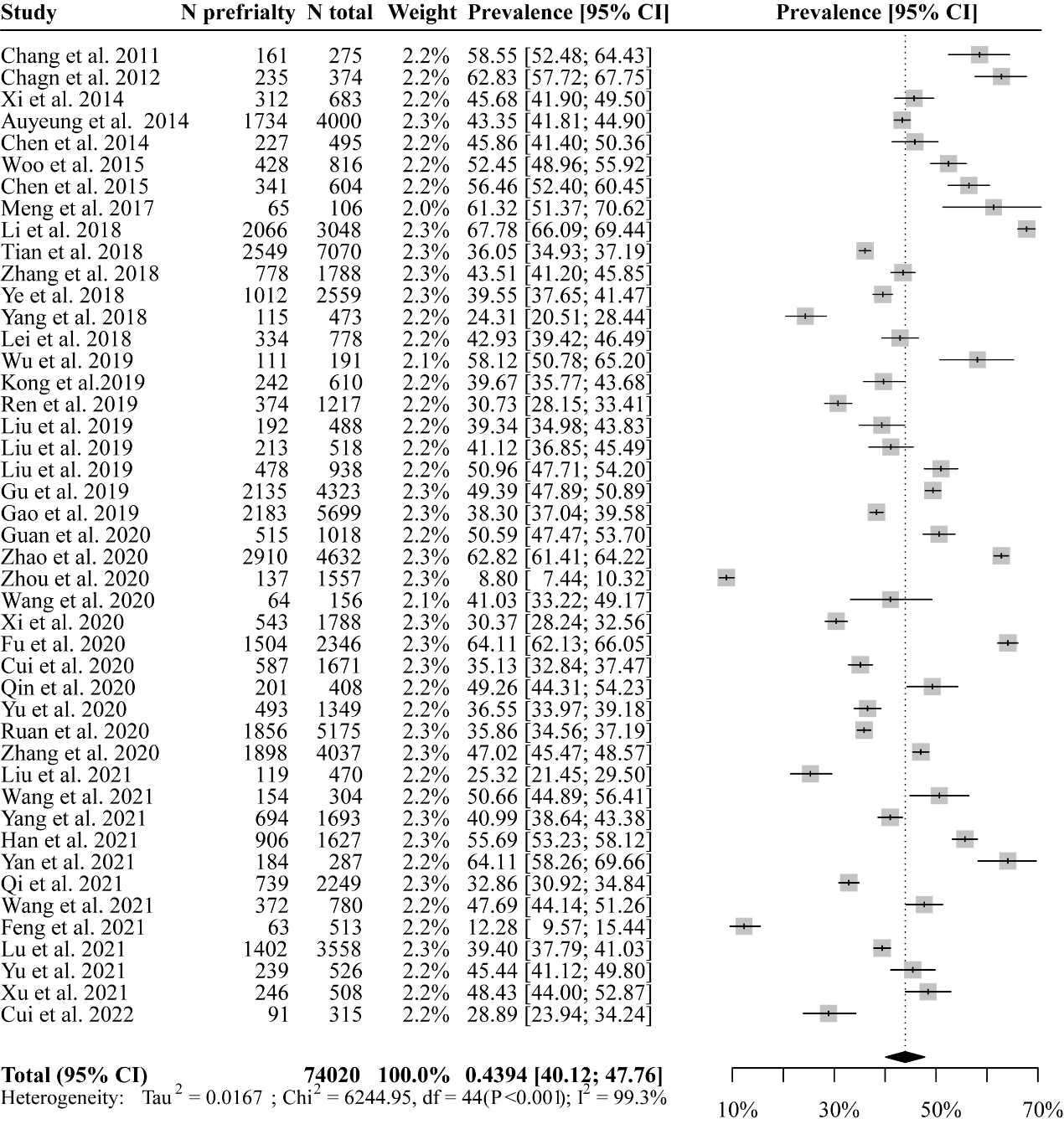


**Supplementary material 6** Mean gross domestic product (GDP) and its association with frailty. A, GDP for the investigated cities in recent 10 years. The data from 21 cities were downloaded from WIND Database by using its Financial Terminal (<https://www.wind.com.cn>). GDP conditions were ranked (high, middle, and low groups) according to the tertiles of the mean GDP. B, The prevalence of frailty among cities with high, middle, and low economic development.

**
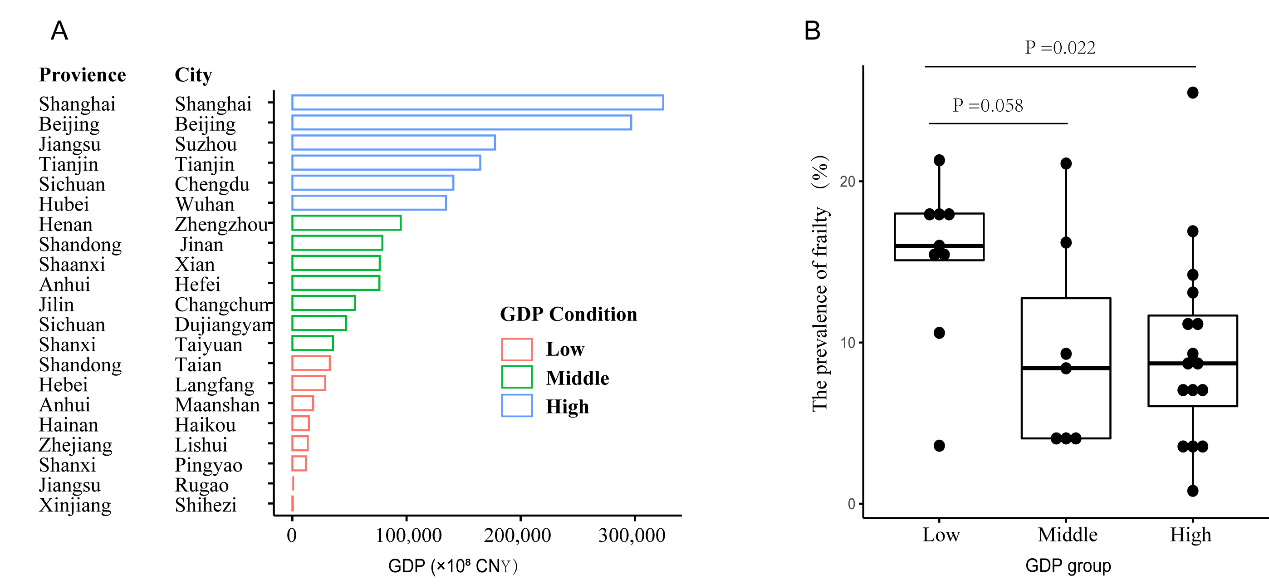
**

**Supplementary material 7** Linear regressions for the association between frailty prevalence and the mean gross domestic product (GDP).

| Model | GDP | Effect size | P for GDP | R^2^ | P trend |
| --- | --- | --- | --- | --- | --- |
| Model 1 |  |  |  |  |  |
|  | Middle | -5.2 | 0.11 | 0.14 | 0.04 |
|  | High | -5.3 | 0.05 |  |  |
| Model 2 |  |  |  |  |  |
|  | Middle | -5.2 | 0.11 | 0.13 | 0.03 |
|  | High | -5.8 | 0.03 |  |  |
| Model 3 |  |  |  |  |  |
|  | Middle | -5.0 | 0.12 | 0.24 | 0.03 |
|  | High | -5.8 | 0.03 |  |  |

Model 2 was adjusted for age. Model 3 was adjusted for age and sex. Cities at high, middle, and low GDP conditions were defined based on tertiles of their GDP data. A total of 21 cities (Supplementary material 5) in 31 studies were included in the regression. Cites at low GDP level (Low group) were taken as the reference.

**Supplementary material 8** Sensitive analysis for the associations between frailty and schooling time according to age, sex, urbanity, sample size, and GDP level.

|  | Study  number | Participant  number | Odds ratio*  (95%CI) | I^2^(%) |
| --- | --- | --- | --- | --- |
| Age |  |  |  |  |
| <70 years | 6 | 19,881 | 1.45(1.03-1.82) | 17.6 |
| ≥70 years | 14 | 12741 | 1.62(1.25-2.08) | 59.2 |
| Percent of females |  |  |  |  |
| < 50% | 7 | 4,914 | 1.88(1.41-2.50) | 25.6 |
| ≥50% | 18 | 41,757 | 1.76(1.38-2.24) | 85.5 |
| Percentage of urbanity |  |  |  |  |
| Urban city | 7 | 6017 | 1.45(0.89 -2.36) | 70.1 |
| Rural area | 4 | 6638 | 1.62(0.91-2.89) | 73.3 |
| Sample size |  |  |  |  |
| <1000 | 12 | 5,873 | 1.65(1.22-2.24) | 46.9 |
| ≥1000 | 13 | 40,798 | 1.87(1.45-2.41) | 88.4 |
| GDP |  |  |  |  |
| Low | 4 | 8,942 | 1.55 (0.93-2.59) | 64.9 |
| Middle and High | 13 | 15,706 | 1.81(1.31-2.51) | 76.5 |

^*^ Participants who had schooling time less than 6 years were taken as the reference. Random-effect meta-analysis was performed for each subgroup. GDP, gross domestic product. I^2^, residual heterogeneity/unaccounted variability.

**Supplementary material 9** Subgroup analysis for the associations between frailty and marriage according to age, sex, schooling time, urbanity, and GDP level.

|  | Study  number | Participant  number | Odds ratio*  (95%CI) | I^2^(%) |
| --- | --- | --- | --- | --- |
| Age |  |  |  |  |
| <70 years | 6 | 20,796 | 0.61(0.44-0.86) | 89.1 |
| ≥70 years | 12 | 22,768 | 0.66(0.50-0.87) | 80.8 |
| Percent of people with less than 6 years of education |  |  |  |  |
| < 50% | 10 | 21,131 | 0.83(0.56-1.23) | 61.8 |
| ≥50% | 13 | 31032 | 0.52(0.39-0.69) | 92.0 |
| Percent of females |  |  |  |  |
| < 50% | 6 | 7,716 | 0.64(0.41-1.01) | 85.7 |
| ≥50% | 17 | 39,827 | 0.59(0.49-0.73) | 78.8 |
| Percentage of urbanity |  |  |  |  |
| Urban city | 6 | 7,968 | 0.76(0.50-1.17) | 73.0 |
| Rural area | 5 | 9,889 | 0.73(0.57-0.93) | 63.6 |
| Sample size |  |  |  |  |
| <1000 | 9 | 5,197 | 0.77(0.52-1.16) | 57.7 |
| ≥1000 | 14 | 42,346 | 0.60(0.48-0.74) | 87.2 |
| GDP |  |  |  |  |
| Low | 5 | 13,211 | 0.80(0.60-1.06) | 87.6 |
| Middle and High | 13 | 18,789 | 0.68(0.53-0.86) | 61.5 |

^*^ Unmarried participants were taken as the reference. Random-effects meta-analysis were performed for each subgroup. GDP, gross domestic product. I^2^, residual heterogeneity/unaccounted variability.

**Supplementary material 10** Subgroup analysis for the associations between frailty and solidary living according to age, sex, educational time, urbanity, and GDP level.

|  | Study  number | Participant  number | OR^*^ (95%CI) | I^2^(%) |
| --- | --- | --- | --- | --- |
| Age |  |  |  |  |
| <70 years | 3 | 9334 | 1.60(0.88-2.91) | 88.1 |
| ≥70 years | 11 | 10,106 | 1.40 (0.79-2.49) | 89.8 |
| Percent of females |  |  |  |  |
| < 50% | 5 | 7248 | 0.74(0.34-1.58) | 68.2 |
| ≥50% | 10 | 12964 | 1.81 (1.12-2.91) | 89.2 |
| Percent of people with less than 6 years of education |  |  |  |  |
| < 50% | 7 | 7446 | 0.86 (0.61-1.21) | 75.0 |
| ≥50% | 5 | 8008 | 1.75 (0.64-4.78) | 93.9 |
| Percentage of urbanity |  |  |  |  |
| Urban city | 8 | 10163 | 1.14 (0.79-1.66) | 61.8 |
| Rural area | 2 | 3997 | 1.89(1.47-2.43) | 0 |
| Sample size |  |  |  |  |
| <1000 | 7 | 3.190 | 0.82(0.42-1.61) | 72.3 |
| ≥1000 | 8 | 17,022 | 1.88(1.14-3.11) | 93.0 |
| GDP |  |  |  |  |
| Low | 3 | 7,555 | 1.39(0.86-2.25) | 85.6 |
| Middle and High | 12 | 12,657 | 1.33(0.76-2.34) | 88.2 |

^*^ Non-solitaries were taken as the reference. Random-effects meta-analysis were performed in each subgroup. GDP, gross domestic product. I^2^, residual heterogeneity/unaccounted variability.

**Supplementary material 11** Meta-regression for the prevalence of frailty. Ten models were conducted in meta-regression. Nine univariate models (from Model 1 to Model 9) were used to find a potential moderator for the prevalence of frailty, and then a multivariate model (Model 10) were conducted by combing the potential moderators. Age, GDP level, and urbanity were considered as important moderators (all *P* < 0.05, all R^2^ > 5%). All the regressions were performed according to a mixed-effects model using a R package “metafor”.

| **Model** | **Covariates** | Study  number | **Coefficient** | **P for coefficients** | **R^2^** | **I^2^(%)** |
| --- | --- | --- | --- | --- | --- | --- |
| Model 1 | Age (years) | 43 | 0.007 | 0.01 | 11.4% | 99.3 |
| Model 2 | Percent of females (%) | 53 | 0.028 | 0.31 | 0 | 99.5 |
| Model 3 | Percent of solitary (%) | 17 | -0.32 | 0.49 | 0 | 99.6 |
| Model 4 | Percent of married (%) | 26 | -0.25 | 0.09 | 7.0% | 99.6 |
| Model 5^a^ | Urban (yes, no) | 26 | -0.057 | 0.04 | 10.3% | 99.0 |
| Model 6 | Sample size (<1000, ≥1000) | 32 | 0.0107 | 0.64 | 0 | 99.5 |
| Model 7**^b^** | GDP | 41 |  |  | 23.3% | 99.3 |
|  | Middle level |  | -0.0891 | 0.009 |  |  |
|  | High level |  | -0.0982 | 0.003 |  |  |
| Model 8**^c^** | Language (Chinese, English) | 53 | -0.006 | 0.792 | 0 | 99.5 |
| Model 9 | Schooling≤6 years (%) | 50 | 0.01 | 0.65 | 0 | 99.5 |
| Model 10**^c^** | Age + GDP + Urbanity | 20 |  |  | 12.8% | 98.8 |
|  | Age |  | 0.010 | 0.09 |  |  |
|  | GDP middle level |  | -0.0175 | 0.70 |  |  |
|  | GDP high level |  | -0.0443 | 0.37 |  |  |
|  | Urban |  | -0.0510 | 0.27 |  |  |

^a, b, c^ Rural areas, Cities with GDP at low levels, and the “Chinese” variable were taken as the reference in the three models, respectively. R^2^: the amount of heterogeneity accounted for. I^2^, residual heterogeneity/unaccounted variability.

**Supplementary material 12** Funnel plot for assessing publication or other types of biases in the meta-analysis of the prevalence of frailty (A) and prefrailty (B). Egger's weighted regression test were used to confirm the funnel plot asymmetry. The double-arcsine-transformed prevalence was used for frailty because some small values may affect the Egger’s weighted regression test.


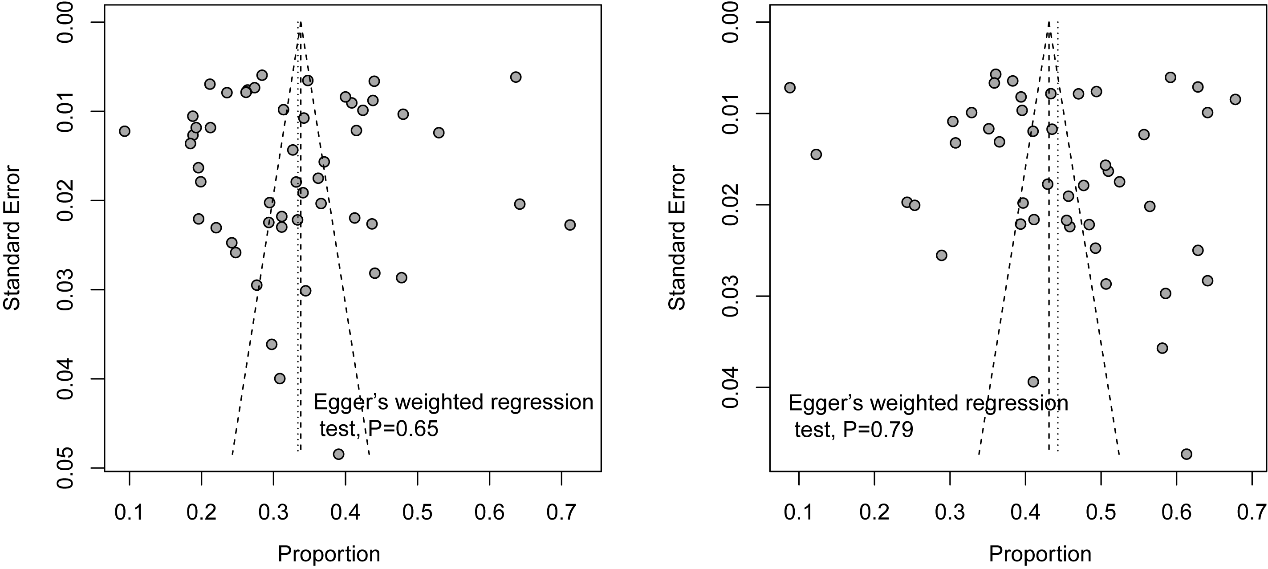


**PRISMA_2020_checklist**

| **Section and Topic** | **Item #** | **Checklist item** | **Location where item is reported** |
| --- | --- | --- | --- |
| **TITLE** | | |  |
| Title | 1 | Identify the report as a systematic review. | See the Title |
| **ABSTRACT** | | |  |
| Abstract | 2 | See the PRISMA 2020 for Abstracts checklist. | See the Abstract |
| **INTRODUCTION** | | |  |
| Rationale | 3 | Describe the rationale for the review in the context of existing knowledge. | See the Introduction，the Paragraph 3 |
| Objectives | 4 | Provide an explicit statement of the objective(s) or question(s) the review addresses. | See the Introduction，the Paragraph 4 |
| **METHODS** | | |  |
| Eligibility criteria | 5 | Specify the inclusion and exclusion criteria for the review and how studies were grouped for the syntheses. | See the Methods, “Search strategy and study selection”, the paragraph 5 |
| Information sources | 6 | Specify all databases, registers, websites, organisations, reference lists and other sources searched or consulted to identify studies. Specify the date when each source was last searched or consulted. | See the Methods, “Search strategy and study selection”, the paragraph 1 |
| Search strategy | 7 | Present the full search strategies for all databases, registers and websites, including any filters and limits used. | See the Methods, “Search strategy and study selection”, the paragraph 2 |
| Selection process | 8 | Specify the methods used to decide whether a study met the inclusion criteria of the review, including how many reviewers screened each record and each report retrieved, whether they worked independently, and if applicable, details of automation tools used in the process. | See the Methods, “Search strategy and study selection”, the paragraph 3 |
| Data collection process | 9 | Specify the methods used to collect data from reports, including how many reviewers collected data from each report, whether they worked independently, any processes for obtaining or confirming data from study investigators, and if applicable, details of automation tools used in the process. | See the Methods, “Data extraction”, the paragraph 1. |
| Data items | 10a | List and define all outcomes for which data were sought. Specify whether all results that were compatible with each outcome domain in each study were sought (e.g. for all measures, time points, analyses), and if not, the methods used to decide which results to collect. | See the Methods, “Search strategy and study selection”, the paragraph 4-5 |
|  | 10b | List and define all other variables for which data were sought (e.g. participant and intervention characteristics, funding sources). Describe any assumptions made about any missing or unclear information. | See the Methods, “Data extraction”, the paragraph 1-2 |
| Study risk of bias assessment | 11 | Specify the methods used to assess risk of bias in the included studies, including details of the tool(s) used, how many reviewers assessed each study and whether they worked independently, and if applicable, details of automation tools used in the process. | See the Methods, “Data analysis”, the paragraph 1-4 |
| Effect measures | 12 | Specify for each outcome the effect measure(s) (e.g. risk ratio, mean difference) used in the synthesis or presentation of results. | See the Methods, “Data analysis”, the paragraph 1-2 |
| Synthesis methods | 13a | Describe the processes used to decide which studies were eligible for each synthesis (e.g. tabulating the study intervention characteristics and comparing against the planned groups for each synthesis (item #5)). | See the Methods, “Data analysis”, the paragraph 3 |
|  | 13b | Describe any methods required to prepare the data for presentation or synthesis, such as handling of missing summary statistics, or data conversions. | See the Methods, “Data analysis”, the paragraph 3 |
|  | 13c | Describe any methods used to tabulate or visually display results of individual studies and syntheses. | See the Methods, “Data analysis”, the paragraph 2-3 |
|  | 13d | Describe any methods used to synthesize results and provide a rationale for the choice(s). If meta-analysis was performed, describe the model(s), method(s) to identify the presence and extent of statistical heterogeneity, and software package(s) used. | See the Methods, “Data analysis”, the paragraph 3-4 |
|  | 13e | Describe any methods used to explore possible causes of heterogeneity among study results (e.g. subgroup analysis, meta-regression). | See the Methods, “Data analysis”, the paragraph 3 |
|  | 13f | Describe any sensitivity analyses conducted to assess robustness of the synthesized results. | See the Methods, “Data analysis”, the paragraph 3 |
| Reporting bias assessment | 14 | Describe any methods used to assess risk of bias due to missing results in a synthesis (arising from reporting biases). | See the Methods, “Data analysis”, the paragraph 3 |
| Certainty assessment | 15 | Describe any methods used to assess certainty (or confidence) in the body of evidence for an outcome. | See the Methods, “Data analysis”, the paragraph 1 |
| **RESULTS** | | |  |
| Study selection | 16a | Describe the results of the search and selection process, from the number of records identified in the search to the number of studies included in the review, ideally using a flow diagram. | See the Results, “Search results and characteristics of the included studies”, Paragraph 1. |
|  | 16b | Cite studies that might appear to meet the inclusion criteria, but which were excluded, and explain why they were excluded. | See the Results, “Search results and characteristics of the included studies”, Paragraph 2. Figure 1 and Supplementary material 1 |
| Study characteristics | 17 | Cite each included study and present its characteristics. | See the Results, Supplementary material 1 |
| Risk of bias in studies | 18 | Present assessments of risk of bias for each included study. | See the Results, Supplementary material 2 |
| Results of individual studies | 19 | For all outcomes, present, for each study: (a) summary statistics for each group (where appropriate) and (b) an effect estimate and its precision (e.g. confidence/credible interval), ideally using structured tables or plots. | See the Results, Supplementary material 1 |
| Results of syntheses | 20a | For each synthesis, briefly summarise the characteristics and risk of bias among contributing studies. | See the Results, “Meta-analysis of the prevalence of frailty and prefrailty” |
|  | 20b | Present results of all statistical syntheses conducted. If meta-analysis was done, present for each the summary estimate and its precision (e.g. confidence/credible interval) and measures of statistical heterogeneity. If comparing groups, describe the direction of the effect. | See the Results, “Stratified meta-analysis for the prevalence of frailty and prefrailty” and “Relationship between frailty prevalence and city gross domestic product (GDP)” , Table 1 |
|  | 20c | Present results of all investigations of possible causes of heterogeneity among study results. | See the Results, ”Sensitivity analysis, meta-regression, and publication of bias” |
|  | 20d | Present results of all sensitivity analyses conducted to assess the robustness of the synthesized results. | See the Results, ”Sensitivity analysis, meta-regression, and publication of bias” |
| Reporting biases | 21 | Present assessments of risk of bias due to missing results (arising from reporting biases) for each synthesis assessed. | See the Results, ”Sensitivity analysis, meta-regression, and publication of bias” |
| Certainty of evidence | 22 | Present assessments of certainty (or confidence) in the body of evidence for each outcome assessed. | See Figure 2 &3, Table 1 and Table 2 |
| **DISCUSSION** | | |  |
| Discussion | 23a | Provide a general interpretation of the results in the context of other evidence. | See the Discussion, the Paragraph 1 |
|  | 23b | Discuss any limitations of the evidence included in the review. | See the Discussion, the Paragraph 2 and 7 |
|  | 23c | Discuss any limitations of the review processes used. | See the Discussion, the Paragraph 7 |
|  | 23d | Discuss implications of the results for practice, policy, and future research. | See the Discussion, the Paragraph 7 |
| **OTHER INFORMATION** | | |  |
| Registration and protocol | 24a | Provide registration information for the review, including register name and registration number, or state that the review was not registered. | See the Method, “Protocol” |
|  | 24b | Indicate where the review protocol can be accessed, or state that a protocol was not prepared. | See the Method, “Protocol” |
|  | 24c | Describe and explain any amendments to information provided at registration or in the protocol. | See the Method, “Protocol” |
| Support | 25 | Describe sources of financial or non-financial support for the review, and the role of the funders or sponsors in the review. | See the “funding and acknowledgements. |
| Competing interests | 26 | Declare any competing interests of review authors. | See the “Conflicts of interest” |
| Availability of data, code and other materials | 27 | Report which of the following are publicly available and where they can be found: template data collection forms; data extracted from included studies; data used for all analyses; analytic code; any other materials used in the review. | All the data can be found in the Supplementary materials, which has been mentioned in the manuscript. |

*From:*  Page MJ, McKenzie JE, Bossuyt PM, Boutron I, Hoffmann TC, Mulrow CD, et al. The PRISMA 2020 statement: an updated guideline for reporting systematic reviews. BMJ 2021;372:n71. doi: 10.1136/bmj.n71 For more information, visit: <http://www.prisma-statement.org/>
